# Social network interventions for health behaviours and outcomes: A systematic review and meta-analysis

**DOI:** 10.1371/journal.pmed.1002890

**Published:** 2019-09-03

**Authors:** Ruth F. Hunter, Kayla de la Haye, Jennifer M. Murray, Jennifer Badham, Thomas W. Valente, Mike Clarke, Frank Kee

**Affiliations:** 1 UKCRC Centre of Excellence for Public Health (NI)/Centre for Public Health, Queen’s University Belfast, Belfast, United Kingdom; 2 Department of Preventive Medicine, Keck School of Medicine, University of Southern California, Los Angeles, California, United States of America; 3 Northern Ireland Methodology Hub, Centre for Public Health, Queen’s University Belfast, Belfast, United Kingdom; Sun Yat-Sen University, CHINA

## Abstract

**Background:**

There has been a growing interest in understanding the effects of social networks on health-related behaviour, with a particular backdrop being the emerging prominence of complexity or systems science in public health. Social network interventions specifically use or alter the characteristics of social networks to generate, accelerate, or maintain health behaviours. We conducted a systematic review and meta-analysis to investigate health behaviour outcomes of social network interventions.

**Methods and findings:**

We searched eight databases and two trial registries from 1990 to May 28, 2019, for English-language reports of randomised controlled trials (RCTs) and before-and-after studies investigating social network interventions for health behaviours and outcomes. Trials that did not specifically use social networks or that did not include a comparator group were excluded. We screened studies and extracted data from published reports independently. The primary outcome of health behaviours or outcomes at ≤6 months was assessed by random-effects meta-analysis. Secondary outcomes included those measures at >6–12 months and >12 months. This study is registered with the International Prospective Register of Systematic Reviews, PROSPERO: CRD42015023541. We identified 26,503 reports; after exclusion, 37 studies, conducted between 1996 and 2018 from 11 countries, were eligible for analysis, with a total of 53,891 participants (mean age 32.4 years [SD 12.7]; 45.5% females). A range of study designs were included: 27 used RCT/cluster RCT designs, and 10 used other study designs. Eligible studies addressed a variety of health outcomes, in particular sexual health and substance use. Social network interventions showed a significant intervention effect compared with comparator groups for sexual health outcomes. The pooled odds ratio (OR) was 1.46 (95% confidence interval [CI] 1.01–2.11; I^2^ = 76%) for sexual health outcomes at ≤6 months and OR 1.51 (95% CI 1.27–1.81; I^2^ = 40%) for sexual health outcomes at >6–12 months. Intervention effects for drug risk outcomes at each time point were not significant. There were also significant intervention effects for some other health outcomes including alcohol misuse, well-being, change in haemoglobin A1c (HbA1c), and smoking cessation. Because of clinical and measurement heterogeneity, it was not appropriate to pool data on these other behaviours in a meta-analysis. For sexual health outcomes, prespecified subgroup analyses were significant for intervention approach (*p* < 0.001), mean age of participants (*p* = 0.002), and intervention length (*p* = 0.05). Overall, 22 of the 37 studies demonstrated a high risk of bias, as measured by the Cochrane Risk of Bias tool. The main study limitations identified were the inclusion of studies of variable quality; difficulty in isolating the effects of specific social network intervention components on health outcomes, as interventions included other active components; and reliance on self-reported outcomes, which have inherent recall and desirability biases.

**Conclusions:**

Our findings suggest that social network interventions can be effective in the short term (<6 months) and longer term (>6 months) for sexual health outcomes. Intervention effects for drug risk outcomes at each time point were not significant. There were also significant intervention effects for some other health outcomes including alcohol misuse, well-being, change in HbA1c, and smoking cessation.

## Abstract 2

Ruth Hunter and colleagues report a systematic review and meta-analysis of behavioural interventions employing social networks, along with outcomes.

## Author summary

**Why was this study done?:**

- Social network interventions specifically use or alter the characteristics of social networks to generate, accelerate, or maintain health behaviours and positive health outcomes.
- Results from previous systematic reviews provided some evidence that social network interventions were effective for improving social support and haemoglobin A1c (HbA1c) outcomes; however, the few studies identified had a high risk of bias.
- The optimal way to apply social network intervention approaches to various health interventions remains unknown.

**What did the researchers do and find?:**

- We conducted a systematic review and meta-analyses of 37 studies investigating the effectiveness of social network interventions for health behaviours and outcomes (or their surrogates).
- Our findings show a significant effect of social network interventions for a range of health behaviours and outcomes, in particular for sexual health outcomes, both in the short and longer term. Subgroup analyses were significant for the intervention approach and when trials were grouped on the basis of mean age and percentage of females.
- In total, 22 out of the 37 studies identified had a high risk of bias, and included studies employed different study designs of variable quality.

**What do these findings mean?:**

- Evidence from this study suggests that social network interventions are associated with positive health behaviours and outcomes.
- Researchers and public health practitioners should consider how to use the social networks of their populations when delivering health behaviour interventions in order to maximise effectiveness.
- We recommend that the scientific community should move beyond individual-level approaches to design and test interventions that use the largely untapped potential of social networks to improve health behaviours and outcomes.

## Introduction

Social networks of family, friends, neighbours, work colleagues, acquaintances, and others have significant impact on our health, health behaviours [1–4], and our ability to change behaviours. However, even though these networks are pervasive in the course of daily life, they have seldom been harnessed in studies of health behaviour interventions [5,6]. Most existing interventions continue to focus on individual-level behaviour and beliefs and fail to address the influential role of an individual’s social systems and environments. In recent years, there has been a growing interest in understanding the effects of social networks on health behaviour, which has been accelerated by the emerging prominence of complexity or systems science in public health [7].

Significant developments in our understanding of the structure, characteristics, and function of social networks, and the impact they have on health, have provided opportunities for novel interventions to improve the health of individuals, communities, and populations. Social network interventions specifically use or alter the characteristics of social networks to generate, accelerate, or maintain health behaviours and positive health outcomes [8]. Such approaches have the potential to support various types of health promotion efforts (e.g., health communication, family, or organisational approaches) and to increase the reach or enhance the effectiveness of existing interventions. A landmark paper by Valente (2012) [8] set out a taxonomy of social network intervention approaches. Four approaches were detailed: (1) those that engage individuals who are selected on the basis of some network property and who may have greater roles in providing information or support within their network (see example by Campbell and colleagues [2008] [9]); (2) those that engage certain groups of people (an approach known as segmentation; see example by Buller and colleagues [1999] [10]); (3) those that encourage or enhance peer-to-peer interactions to cascade information and effects to other network members (a process known as induction; see example by Hoffman and colleagues [2013] [11]); and (4) those that involve changing the network (alteration) by adding or deleting members, adding or deleting specific social ties, or changing the entire network (see example by Litt and colleagues [2007] [12]). Such approaches can improve the efficiency and effectiveness of public health interventions because they leverage important mechanisms for behaviour change (e.g., the influence of social norms, social learning, and social support) [13] and potentially enhance behaviour change maintenance [14].

Although research in social networks dates back to the 1930s, social networks are not routinely considered in public health interventions. Extant research has largely focused on observational [1–4] and simulation studies [15–17]. Observational studies have identified features of social networks associated with health behaviours or outcomes that may be important targets for interventions, and simulation studies have begun to explore the potential impact of using social network characteristics for behaviour change [17]. However, real-world interventions that use social networks are less common.

Previous systematic reviews and meta-analyses have provided evidence to support the effectiveness of social network interventions for some specific health outcomes. A meta-analysis by Spencer-Bonilla and colleagues (2017) [18] involving 19 randomised controlled trials (RCTs) investigated the effectiveness of social network interventions on social support, glycaemic control, and quality of life in patients with type 2 diabetes. Results demonstrated that interventions improved social support (0.74 SD [95% CI 0.32–1.15]) and haemoglobin A1c (HbA1c) at 3 months (−0.25 percentage points [95% CI −0.40 to −0.11]) but not quality of life. However, the few trials identified had a high risk of bias. A systematic review by Wang and colleagues (2011) [19] focused solely on condom use. Among the nine included studies with control groups, eight showed significant improvements in at least one measure of condom use. Therefore, there is a need to further investigate the effectiveness of social network interventions for a range of other health behaviours and outcomes including drug use, diet, physical activity, screening, vaccinations, etc. There is also a need to explore the impact of different network intervention approaches, as this has not been done in other reviews and would further advance our understanding of how network interventions operate. Also, these previous reviews mostly included dyadic-level approaches involving spouses or pairs of other family members, emphasising the need for a review that focuses on network interventions that move beyond the dyad level.

The explicit use of social network data, which map the structure of social connections among multiple people, distinguishes social network interventions from the large body of general peer support and social support interventions that have been extensively studied and that typically focus on individuals’ perceptions of social phenomena (e.g., social norms) or on dyads [20]. As such, the optimal way to apply the myriad of social network intervention approaches to various health interventions remains unknown. For example, it is not clear who in a social network should be engaged to catalyse the diffusion of behaviour change, nor which mechanisms can best be harnessed to maximise the effects of an intervention, though some have suggested using theory as a guide [6]. The present study addresses this gap through a systematic review and meta-analysis of studies that aimed to harness social network interventions to improve health behaviours and outcomes (or their surrogates). We also examine whether different network interventions approaches—individual, segmentation, induction, or alteration—vary in their effectiveness.

## Methods

### Search strategy and selection criteria

This study is registered with PROSPERO [21] (International Prospective Register of Systematic Review) (CRD42015023541) and is reported according to the Preferred Reporting Items for Systematic Reviews and Meta-Analyses (PRISMA) [22].

We searched Medline, Embase, Web of Knowledge, Scopus, Psychinfo, Education Resources Information Center (ERIC), the International Bibliography of the Social Sciences (IBSS), Sociological Abstracts Trial, the Cochrane Central Register of Controlled Trials (CENTRAL), the World Health Organization (WHO) International Clinical Trials Registry Portal (ICTRP), and ClinicalTrials.gov from 1990 until May 28, 2019.

We included randomised trials and controlled before-and-after studies (i.e., study designs that included a control group [participants were not randomised to groups] and collected data pre- and postintervention) that compared a social network intervention (whether given alone or in combination with other intervention components) against the following comparators: usual care, no intervention, waiting-list control, or an intervention with no explicit social network component. We included studies that addressed all age groups regardless of health status but limited the studies to those reported in English. Reference lists of relevant studies were also screened. Details of methods and the search strategies are described in S1 Text.

### Data extraction and quality assessment

Two authors (RFH and KdlH) independently screened the titles and abstracts of retrieved citations to identify potentially relevant studies. The full articles were evaluated if a decision could not be made based on the titles and abstracts. Relevant data were extracted by two reviewers (RFH and JB) using a standardised form and cross-checked. Any discrepancies were resolved by consensus. The extracted data included study characteristics, participant characteristics, interventions, social network functions (see S1 Text), outcomes, and other relevant findings. The Cochrane Risk of Bias assessment tool [23] was used to assess risk of bias (see S1 Text). When publications lacked sufficient detail for full data extraction, we contacted the original authors for the necessary information.

### Types of interventions

Social network interventions were defined as those that purposefully used social networks or social network data to generate social influence and/or accelerate behaviour change among individuals, communities, organisations, or populations [8]. Interventions could use existing networks, establish new networks, disrupt harmful networks, or use social network data to educate participants about the potential influences of their health behaviours on their network members, including in vivo and online social networks. A broad definition of ‘social networks’ was employed to encompass social interactions and personal relationships [24], such as with friends, school friends, nonschool friends, family members, sexual partners, work colleagues, neighbours, and networks in which an unhealthy behaviour is shared (such as substance misuse and risky sex practices). The study must have measured social relationships, and that relationship must have been used in some aspect of the intervention design or delivery. The measurement of relationships must have been beyond the dyad level and included ties among multiple actors (i.e., interventions that targeted interactions between spouses or a pair of friends were excluded). Included interventions must have targeted change in health behaviours or health outcomes (or their surrogates). Details of interventions are described in S1 Text. Briefly, individual network interventions included those that specifically used network data to identify certain individuals to be recruited to act as proponents of behaviour change on the basis of some network property. Segmentation network interventions included interventions directed towards groups of people clustered in a network. Induction network interventions involve excitation and activation of existing social ties in a social network to diffuse information or healthy behaviours. Alteration network interventions involve changing the structure of the network by the addition of new members or breaking existing ties with those who foster and facilitate unhealthy, risky behaviours.

### Outcomes

Included studies had a primary outcome of health-related behaviour change using objective or self-report measures (e.g., change in physical activity) or change in a health outcome or relevant proxy/surrogate measure (e.g., diffusion of health promotion information). As well as measures of health outcomes and health behaviours, the study also included behavioural surrogates (e.g., HbA1c) or network surrogates (e.g., reproductive ratio). Details of outcomes are described in S1 Text. A wide range of outcome measures were used in the studies, in keeping with the range of topics investigated. The outcomes related to sexual health, drug risk, weight loss, diet, physical activity, smoking cessation, alcohol/other substance misuse, well-being, change in diabetes marker (HbA1c), mammography screening, ticket redemption for water purification, and reproductive ratio for number of participants installing an app.

### Data synthesis and statistical analysis

Statistical analyses were done in accordance with our registered protocol (PROSPERO: CRD42015023541) [21]. We conducted meta-analyses according to time point of outcome measurement (≤6 months, 6–12 months, last follow-up) for sexual health outcomes (i.e., percent engaging in condomless sex) and drug risk outcomes (i.e., percent engaging in injection drug risk or other drug risk behaviours). Because of clinical and statistical heterogeneity, we were not able to pool data for other outcomes. Log odds ratios (ORs) and standard errors (SEs) were calculated for each study to provide the odds of achieving a more favourable outcome for the intervention group compared with the control group. When studies reported event (numbers or percentages of participants) and denominators data (*k* = 21) or adjusted ORs and 95% confidence intervals (CIs) (*k* = 6), these were used to directly compute log ORs and SEs. When studies reported ORs and *p*-values, *p*-values were converted to SEs using procedures outlined in the Cochrane handbook (*k* = 1). When studies reported means and SDs, standardised mean differences (SMDs) and SEs were calculated and converted to log ORs and SEs using the Chinn (2000) equation [25] (*k* = 12). When studies reported data separately for multiple intervention groups or subgroups, data were combined using procedures outlined in the Cochrane handbook [26] (*k* = 5). All data were transformed so that higher OR values indicated higher odds of achieving a more favourable outcome for intervention groups compared with controls. A significant intervention effect was determined when the 95% CI excluded 1 for the OR. Separate meta-analyses were carried out for studies reporting (1) sexual health outcomes and (2) drug risk outcomes.

A direct meta-analysis was used to pool ORs using a random-effects model. Heterogeneity was assessed using the Cochran Q test and the I^2^ statistic. Heterogeneity and reporting bias were assessed visually using forest and funnel plots created using Stata [27]. The Egger and colleagues’ (1997) [28] and precision-effect estimate with standard error (PEESE) [29] tests for study size effects were used to formally test for publication bias.

We did prespecified subgroup analyses to explore heterogeneity by showing how effect sizes differed between groups of studies. Characteristics included intervention approach (individual, segmentation, induction, alteration), intervention length (≤3 months, 3–6 months, 6–12 months, 12–18 months, >18 months), age of participants (above or below the overall mean age of 32.4 years across studies), and gender of participants (above or below the mean of 45.5% female across studies). Subgroup analyses were done separately for pooled sexual health outcomes and drug risk outcomes.

We conducted prespecified sensitivity analyses to determine whether the results of the meta-analysis were robust to omission of studies classified at high risk of bias using the Cochrane Risk of Bias tool, studies not performing intention-to-treat (ITT) analyses, studies with attrition rates higher than 20%, and studies using nonrandomised designs. Sensitivity analyses were conducted separately for pooled sexual health outcomes and drug risk outcomes.

A significant intervention effect was determined when the 95% CI for the OR excluded 1.00. Statistical significance was set at the 5% level (*p* < 0.05). We used Stata release 13 (StataCorp, College Station, TX, United States) [27] for the analyses.

Apart from the additional sensitivity analysis excluding studies using nonrandomised designs, the study was carried out per the prespecified protocol. The subgroup analyses were prespecified prior to commencement of the review and after registration.

## Results

**Fig 1:**
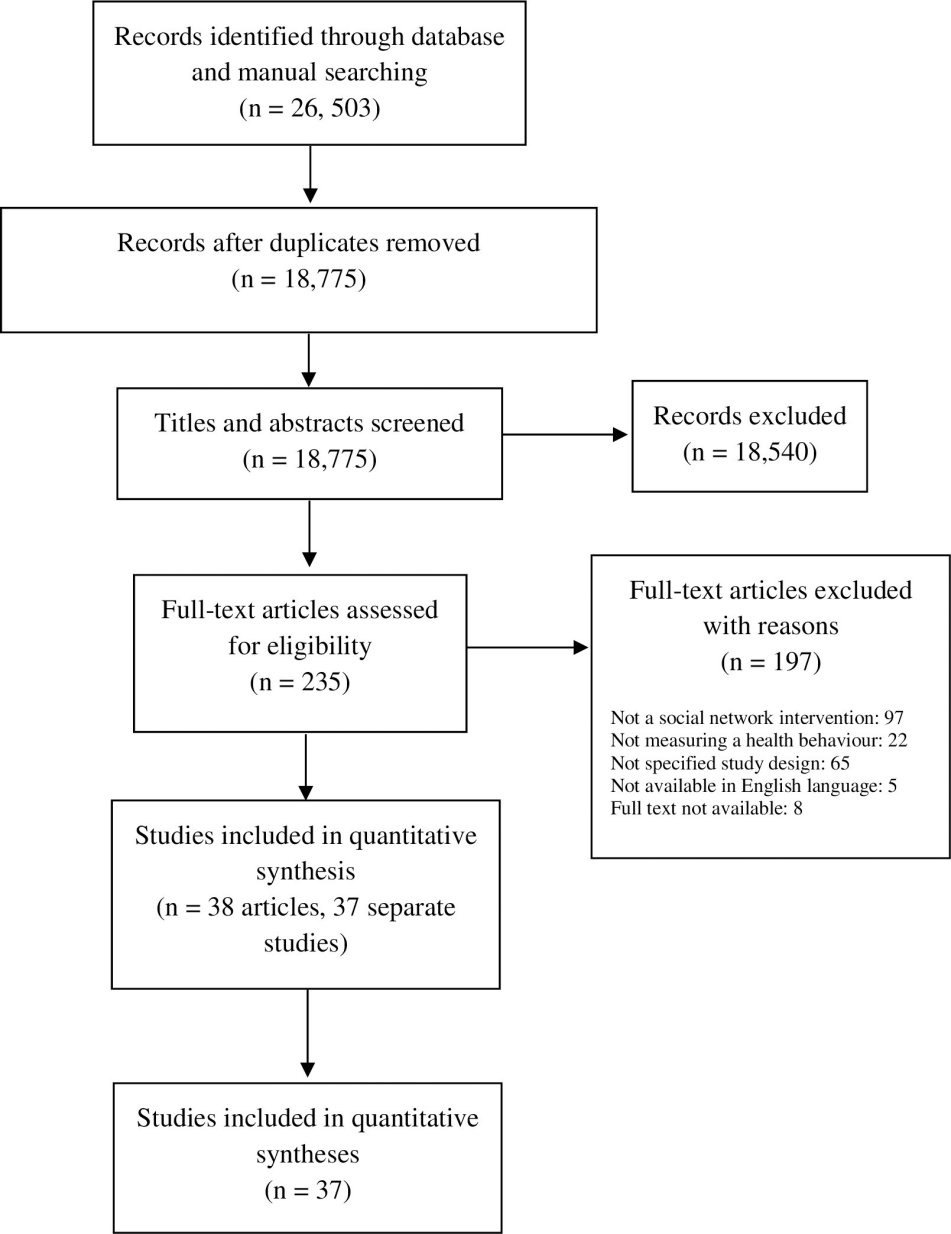
PRISMA flow diagram. PRISMA, Preferred Reporting Items for Systematic Reviews and Meta-Analyses.

We identified 26,503 records, in which 235 potentially eligible articles were reviewed in full text. Of these, 197 were excluded because they did not investigate a social network intervention (*n* = 97), measure a health behaviour or health outcome (*n* = 22), or use our prespecified study design criterion (*n* = 65). This left 37 eligible studies for inclusion in our review. The details of our literature search are reported in Fig 1, which shows the PRISMA flow diagram. Table 1 presents a summary of the characteristics of included studies. Further study characteristics include social network functions (S1–S4 Tables), risk of bias assessment (S1 Fig), and citations for all included studies (S2 Text).

**Table 1:** Trials of social network interventions meeting the inclusion criteria.

Reference	Country	Study Design	Population	Intervention	Control	Outcome Measure(s)	≤6 months OR (95% CI)	>6 months to ≤12 months OR (95% CI)	Last Follow-up OR (95% CI)	Risk of Bias Summary
**Individual Approach**										
Kelly and colleagues, 1997 [1]	USA	RCT	MSM; mean age 31 years	265	173	Mean number of times engaged in UAI during past 2 months	-	1.62 (0.97–2.71)	-	Low
Latkin and colleagues, 1998 [2]	USA	Controlled before and after	Unemployed inner-city PIDs; aged 25–40 years	*n* = 41 peer leaders; *n* = 78 network members	70	% always cleaning used needle	4.68 (2.20–9.96)	-	-	High
Sikkema and colleagues, 2000 [3]	USA	RCT	Women in low-income, inner-city housing; mean age 35.9 years	351	339	Mean % women reporting any UI in past 2 months	-	1.43 (1.06–1.94)	-	High
Amirkhanian and colleagues, 2005 [4]	Russia and Bulgaria	RCT	Young MSM; mean age 23 years	133	143	% reporting any UI	2.49 (1.49–4.16)	1.62 (0.97–2.71)	-	High
Kelly and colleagues, 2006 [5]	Bulgaria	RCT	Roma men; mean age 20 years	145	137	Occurrence of UI during previous 3 months	1.58 (0.94–2.66)	2.39 (1.30–4.38)	-	High
Campbell and colleagues, 2008 [6]	United Kingdom	Cluster RCT	12–13-year-old students	5,358	5,372	Prevalence of smoking in the past week in school-year group	1.31 (1.13–1.52)	1.25 (1.11–1.40)	1.19 (1.08–1.31)	Low
Kim and colleagues, 2015 [7]	*Honduras	Cluster RCT	Members of local village; mean age 35 years	3,740	1,599	Proportions of available products redeemed (product adoption) by population under each targeting method	1.05 (0.95–1.17)	-	-	Low
Amirkhanian and colleagues, 2015 [8]	Russia and Hungary	RCT	MSM; mean age 27–29 years	339	287	Proportion of any UAI in past 3 months	2.17 (1.56–3.02)	1.68 (1.21–2.33)	-	High
Woudenberg and colleagues, 2018 [9]	The Netherlands	Cluster RCT	Healthy adolescents; mean age 12.17 years	118	120	Mean steps per day (Fitbit Flex)	0.90 (0.54–1.51)	-	-	Low
**Segmentation Approach**										
Trotter and colleagues, 1996 [10]	USA	RCT	PIDs or crack smokers; 31% aged 18–24 years	189	89	Composite drug risk (frequency used crack, injected drugs, unbleached needle use); shared cotton, cookers, and/or rinse water	0.80 (0.36–1.74)	-	-	High
Kincaid and colleagues, 2000 [11]	*Bangladesh	Controlled before and after	Community-based females; mean age 30 years	107	753	% beginning contraception use/continuing contraception use	-	-	7.88 (4.94–12.57)	High
Minnis and colleagues, 2014 [12]	USA	Cluster RCT	Latino neighbourhood; mean age 17 years	79	83	Unprotected sex at last sex	2.38 (0.80–7.11)	-	-	High
Shaya and colleagues, 2014 [13]	USA	Partial RCT	Majority African American population with type 2 diabetes; mean age 53 years	68	70	Changes in HbA1c and blood glucose	3.61 (1.93–6.75)	-	-	Low
Cobb and colleagues, 2014 [14]	USA	Randomised, placebo-controlled, parallel-group trial	Healthy adults; mean age 47 years	752	751	Overall well-being measured by the Individual-Level Well-Being Assessment and Scoring Method (scale: 0–100)	1.40 (1.11–1.77)	-	-	High
**Induction Approach**										
Kegeles and colleagues, 1996 [15]	USA	RCT	MSM; mean age 23 years	159	109	Proportion engaging in any UAI in the past 2 months with men, boyfriends/lovers, nonprimary partners	-	1.54 (0.84–2.82)	-	Low
Latkin and colleagues, 1996 [16]	USA	Controlled before and after	PIDs; median age 40 years	39	50	Frequency of needle sharing with HIV-positive and HIV-negative partners	-	-	1.29 (0.66–2.53)	High
Buller and colleagues, 1999 [17]	USA	RCT	Blue-collar employees; mean age 42 years	395	371	Daily fruit and vegetable intake using 24-hour recall questionnaire	-	-	1.28 (0.98–1.67)	Low
Wing and Jeffrey, 1999 [18]	USA	RCT	Healthy adults; mean age 43 years	128	38	Overall weight loss (months 0–4 and months 0–10)	1.89 (0.91–3.92)	2.05 (0.97–4.33)	-	High
Elford and colleagues, 2001 [19]	UK	Controlled before and after	Gay men; median age 33 years	1,646	223	% reported UAI in last 3 months	0.76 (0.52–1.11)	0.85 (0.57–1.28)	0.77 (0.42–1.44)	High
Earp and colleagues, 2002 [20]	USA	Controlled before and after	African American women; 45% aged 50–64 years	438	467	Self-reported mammography in the past 2 years	-	-	0.90 (0.61–1.32)	High
Flowers and colleagues, 2002 [21]	UK	Quasi-experimental, two-by-two, repeat cross-sectional trial	Gay men; mean age 32 years	1,245	1,031	Rate of UAI with casual partners in past year	-	-	1.22 (0.79–1.89)	High
Latkin and colleagues, 2003 [22]	USA	RCT	Low-income African American PIDs; mean age 39 years	167	83	Self-report injection risk behaviours: stopping injection drug use in past 6 months	3.65 (1.23–10.83)	-	-	High
Morisky and colleagues, 2004 [23]	*Philippines	Controlled before and after	Heterosexual male clients of commercial sex workers; mean age 34.7 years	1,819	1,570	Self-reported condom use	-	-	1.36 (1.19–1.56)	High
Garfein and colleagues, 2007 [24]	USA	RCT	PIDs; mean age 23 years	431	423	Self-reported injection behaviours in past 3 months (composite variable)	1.56 (1.07–2.27)	-	-	High
Valente and colleagues, 2007 [25]	USA	Cluster RCT	High-risk adolescents; mean age 16 years	351	534	Change in substance use (cigarettes, alcohol, marijuana, cocaine) (quit rate)	-	1.16 (0.65–2.07)	-	High
Latkin and colleagues, 2009 [26]	USA and *Thailand	RCT	PIDs; 40% aged 40+ years	550	573	Frequency of risk behaviours (injected in last month)	0.70 (0.43–1.12)	-	-	High
Sutcliffe and colleagues, 2009 [27]	*Thailand	RCT	Healthy adults; median age 19 years	495	488	Frequency of methamphetamine use in past 3 months	0.90 (0.67–1.20)	1.09 (0.83–1.43)	-	High
Tobin and colleagues, 2011 [28]	USA	RCT	PIDs; mean age 44 years	114 indexes (163 network members)	113 indexes (173 network members)	Frequency of sharing needles for injection and drug splitting in past 6 months (injection risk)	1.08 (0.53–2.17)	2.13 (1.04–4.35)	2.63 (1.25–5.56)	Low
Bastian and colleagues, 2013 [29]	USA	RCT	Current smokers who were family members/close friends of patients with lung cancer; mean age 47 years	245	251	7-day smoking abstinence	1.20 (0.70–2.06)	0.90 (0.50–1.62)	-	Low
Hoffman and colleagues, 2013 [30]	Russia	RCT	PIDs and their drug and/or sexual network; median age 28 years	99 indexes (127 network members)	92 indexes (114 network members)	Incidence of HIV infection	-	-	2.12 (0.86–5.23)	High
Gotsis and colleagues, 2013 [31]	USA	Randomised crossover	Healthy adults; mean age 36 years	64 (25 ego networks)	78 (29 ego networks)	Self-reported physical activity frequency (single-item measure)	1.46 (0.68–3.17)	-	-	Low
Booth and colleagues, 2016 [32]	*Ukraine	Cluster RCT	PIDs; mean age 32 years	611	589	HIV incidence	-	1.89 (1.41–2.53)	-	Low
Cobb and colleagues, 2016 [33]	USA	RCT (12-cell fractional factorial design)	Adult smokers; mean age 44 years	6,028	3,014	Reproductive ratio: number of individuals installing the app divided by the number of a seed participant’s Facebook friends	-	1.26 (1.10–1.44)	-	Low
**Alteration Approach**										
Wingood and colleagues, 2004 [34]	USA	RCT	Women living with HIV; mean age 35 years	190	176	Self-report UVI	1.10 (0.75–1.61)	1.29 (0.86–1.91)	-	Low
Litt and colleagues, 2007; 2009 [35,36]	USA	RCT	Alcohol dependents; mean age 45 years	140	70	Proportion of days of no alcohol use in past 90 days, number of days of continuous alcohol abstinence for 90 days	3.97 (2.26–6.96)	2.90 (1.66–5.06)	1.77 (1.00–3.13)	High
Eaton and colleagues, 2011 [37]	USA	Randomised efficacy trial	At-risk HIV-negative MSM; mean age 29 years	74	75	Number UAI with HIV-positive or -negative partners	1.08 (0.61–1.94)	-	-	Low
Graham and colleagues, 2016 [38]	USA	RCT randomised, controlled factorial design	Healthy adults; mean age 42 years	2,640	2,650	Website utilisation metrics (number of watched videos on smoking addiction)	0.92 (0.88–0.96)	-	-	Low

References 1–38: see S2 Text. See S1–S4 Tables for further details regarding the network intervention approaches.

- Outcomes not measured or data not available.

* Indicates LMIC as reported in the DAC list of ODA recipients 2019.

Abbreviations: CI, confidence interval; DAC, Development Assistance Committee; HbA1c, haemoglobin A1c; HIV, human immunodeficiency virus; LMIC, low- and middle-income country; MSM, men who have sex with men; PID, person who injects drugs; ODA, Official Development Assistance; OR, odds ratio; RCT, randomised controlled trial; UAI, unprotected anal intercourse; UI, unprotected intercourse; UVI, unprotected vaginal intercourse

The mean age of all participants was 32.4 years (SD 12.7), of whom 24,679 (45.5%) were women (Table 1). The majority of studies addressed human immunodeficiency virus (HIV) prevention behaviours (*n* = 23), including studies involving risky drug use among network partners (e.g., shared needles) and studies of sexual partnership networks. Studies took place mainly in high-income countries (e.g., USA, UK), with only six studies involving low- and middle-income countries (LMICs)—namely, Honduras, Bangladesh, Philippines, Thailand, and Ukraine. The majority of studies predominantly engaged minority populations such as ethnic minority groups (*n* = 5) or men who have sex with men (MSM) (*n* = 7) or those from socio-disadvantaged communities (*n* = 10). Studies typically had an exclusive or prominent focus on one network intervention approach: nine studies used an individuals network intervention approach, five a segmentation approach, 19 an induction approach, and four an alteration approach. The interventions reported in these studies combined behaviour change theories with theories about the influence of networks (see S1–S4 Tables). Behaviour change models included social cognitive theory, theory of reasoned action, theory of planned behaviour, health belief model, social norms theories, social influence, socio-ecological model, social learning theory, and social identity theory. For the network elements, the theoretical underpinning most cited was diffusion of innovation theory.

**Fig 2:**
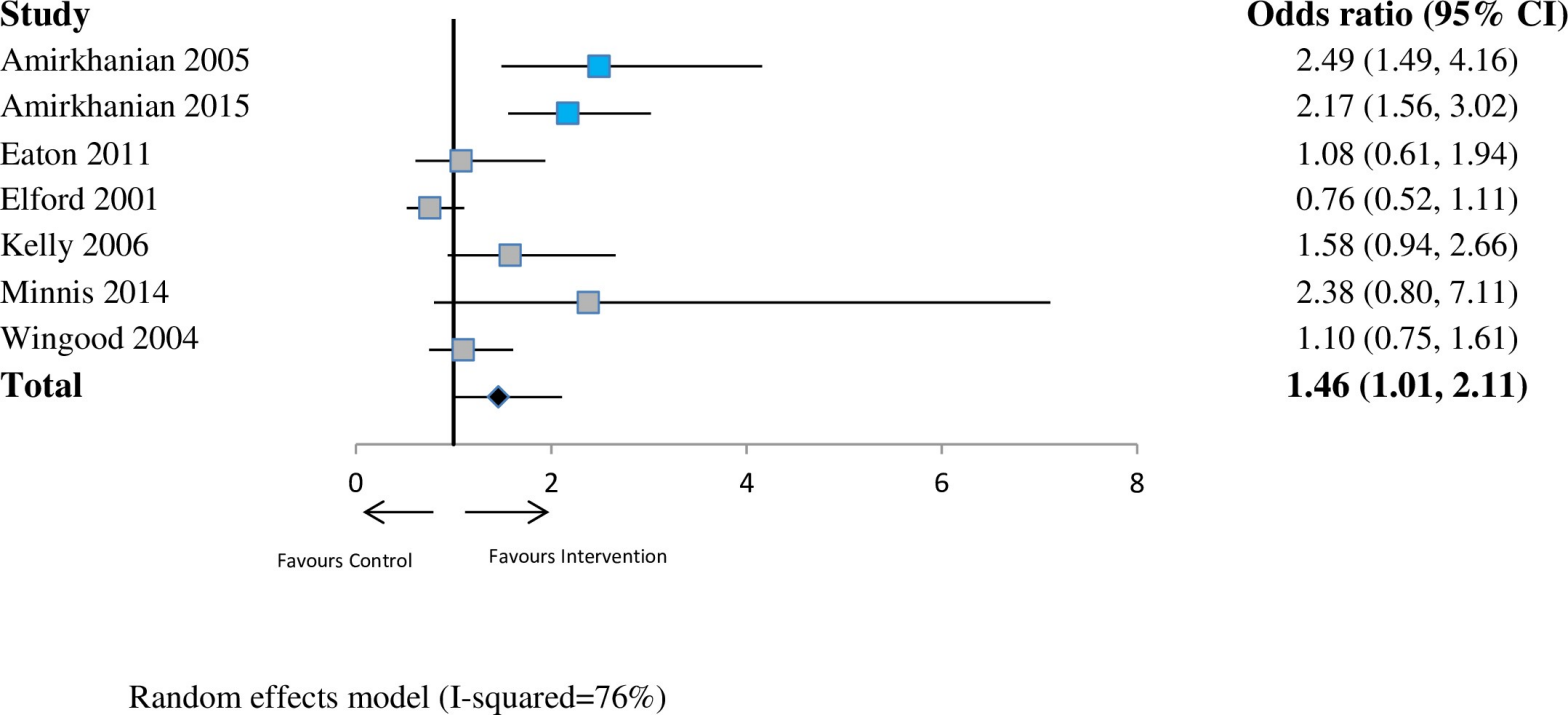
Forest plot showing odds of a more favourable outcome for intervention groups compared with controls for outcomes reported at ≤6 months (sexual health outcome measures). The blue denotes significant effect in favour of the Intervention group. The grey denotes non-significant. The red denotes significant effect in the favour of the control group. CI, confidence interval.

**Fig 3:**
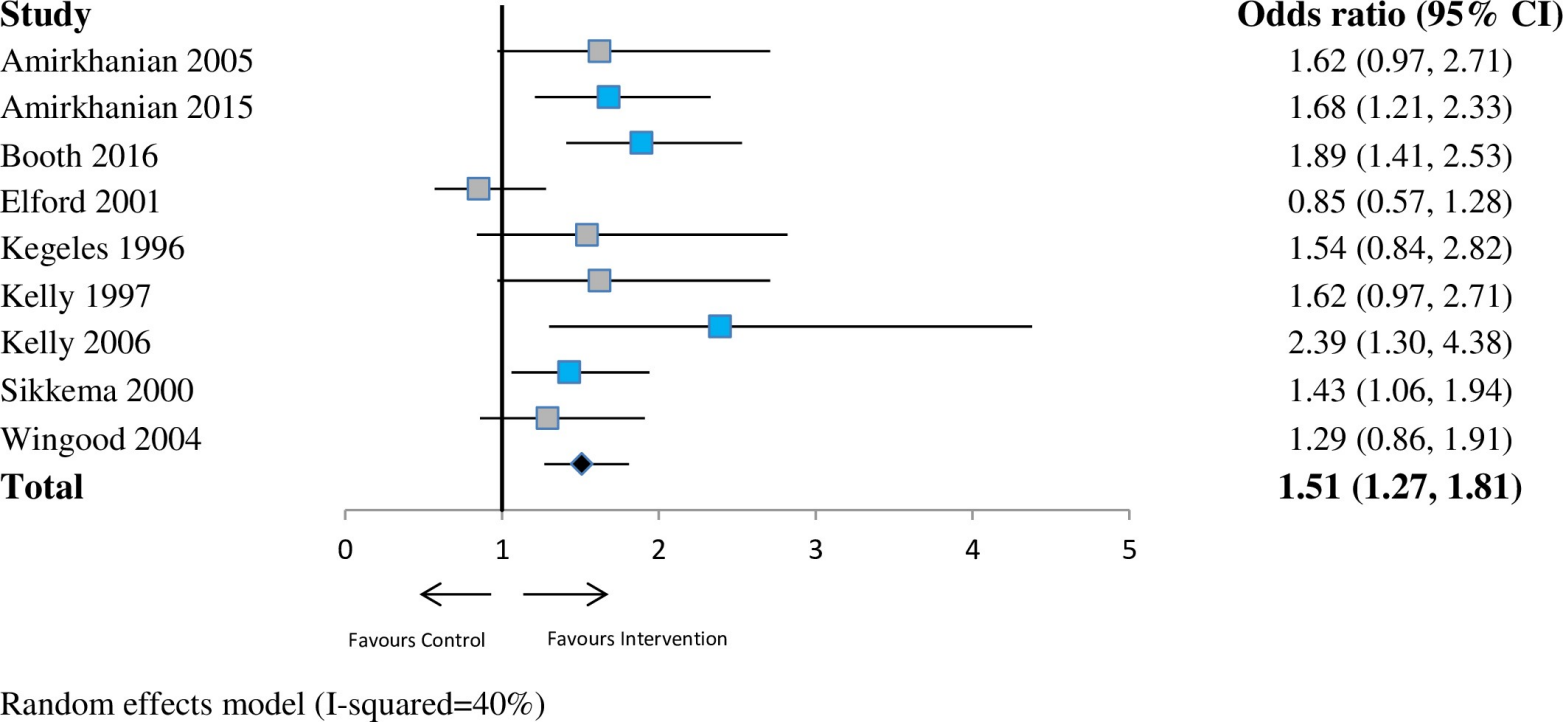
Forest plot showing odds of a more favourable outcome for intervention groups compared with controls for outcomes reported at >6 months to ≤12 months (sexual health outcome measures). The blue denotes significant effect in favour of the Intervention group. The grey denotes non-significant. The red denotes significant effect in the favour of the control group. CI, confidence interval.

**Fig 4:**
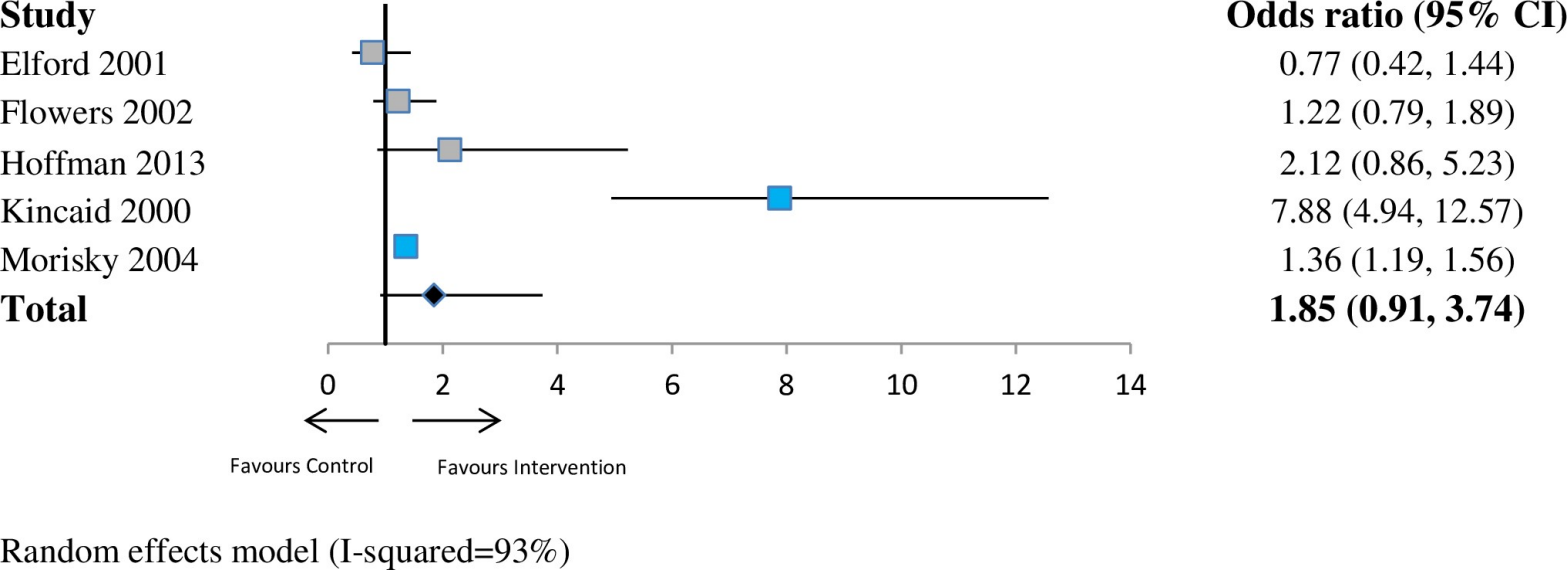
Forest plot showing odds of a more favourable outcome for intervention groups compared with controls for outcomes reported at last follow-up (sexual health outcome measures). The blue denotes significant effect in favour of the Intervention group. The grey denotes non-significant. The red denotes significant effect in the favour of the control group. CI, confidence interval.

For sexual health (*n* = 7 studies), the pooled OR was 1.46 (95% CI 1.01–2.11; *p* < 0.05; I^2^ = 76%) at ≤6 months. This indicates a substantial increase in the odds that network interventions will have a lower prevalence of risky sexual health practices compared with the control group at 6 months. At 6–12 months (*n* = 9 studies), the pooled OR was 1.51 (95% CI 1.27–1.81; *p* < 0.001; I^2^ = 40%). At >12 months (*n* = 5 studies), the pooled OR was 1.85 (95% CI 0.91–3.74; *p* = 0.09; I^2^ = 93%). See Figs 2–4.

**Fig 5:**
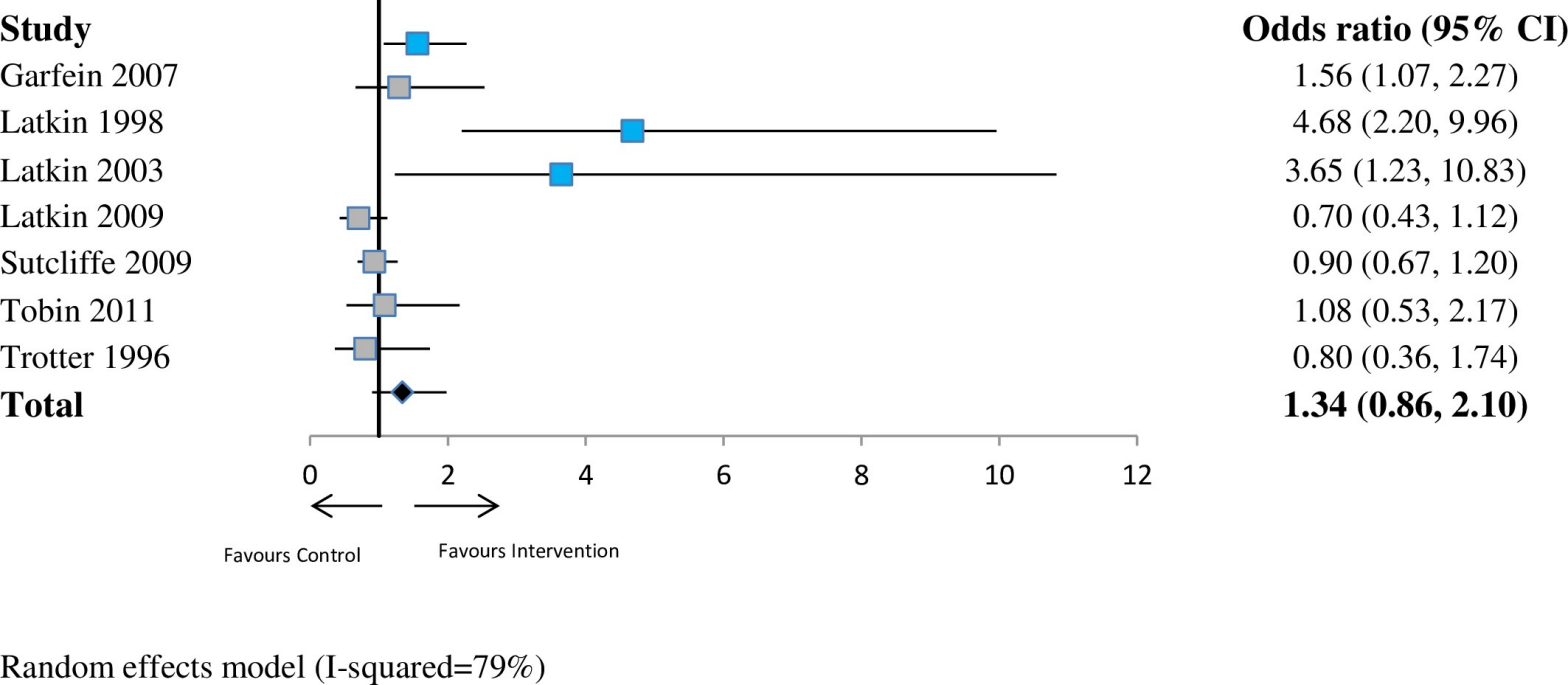
Forest plot showing odds of a more favourable outcome for intervention groups compared with controls for outcomes reported at ≤6 months (drug risk outcome measures). The blue denotes significant effect in favour of the Intervention group. The grey denotes non-significant. The red denotes significant effect in the favour of the control group. CI, confidence interval.

**Fig 6:**
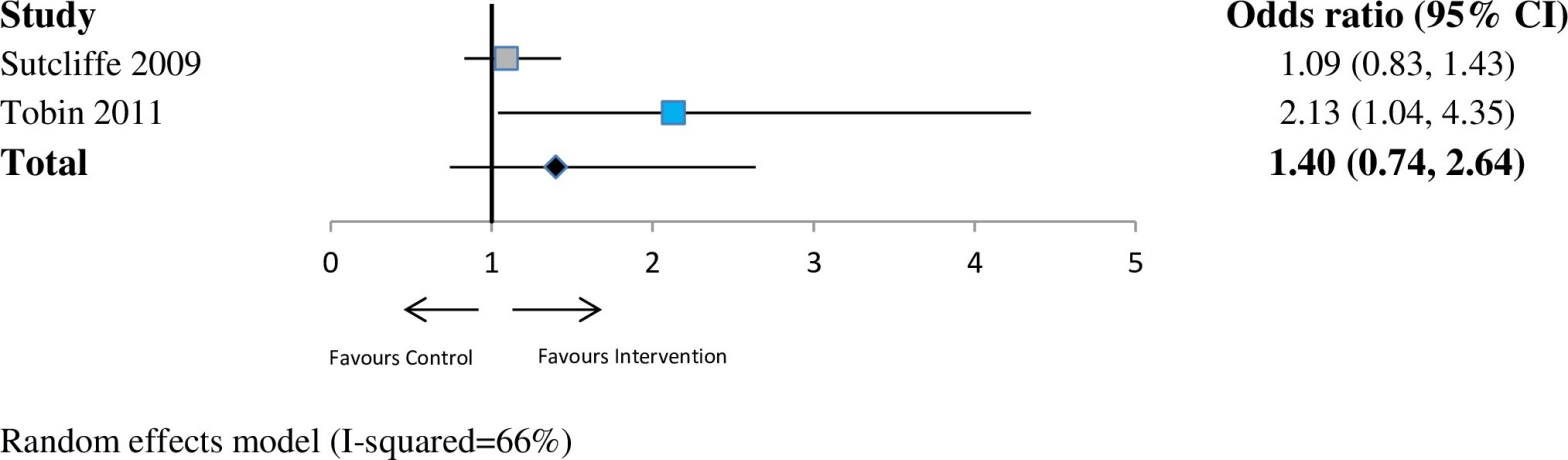
Forest plot showing odds of a more favourable outcome for intervention groups compared with controls for outcomes reported at >6 months to ≤12 months (drug risk outcome measures). The blue denotes significant effect in favour of the Intervention group. The grey denotes non-significant. The red denotes significant effect in the favour of the control group. CI, confidence interval.

**Fig 7:**
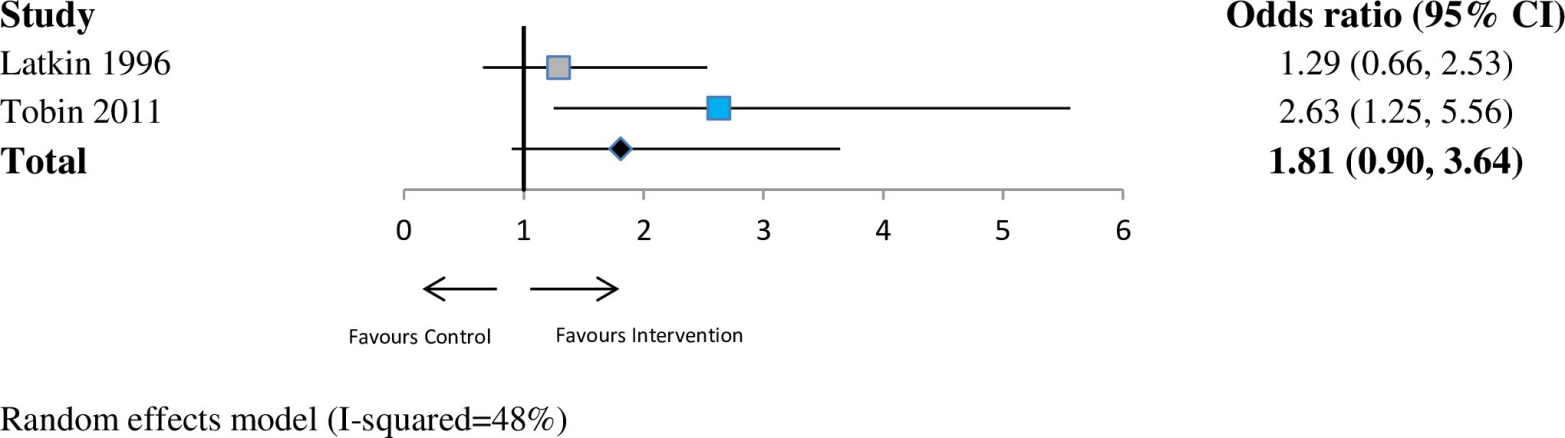
Forest plot showing odds of a more favourable outcome for intervention groups compared with controls for outcomes reported at last follow-up (drug risk outcome measures). The blue denotes significant effect in favour of the Intervention group. The grey denotes non-significant. The red denotes significant effect in the favour of the control group. CI, confidence interval.

For drug risk (*n* = 7 studies), the pooled OR was 1.34 (95% CI 0.86–2.10; *p* = 0.19; I^2^ = 79%) at ≤6 months. At 6–12 months (*n* = 2 studies), the pooled OR was 1.40 (95% CI 0.74–2.64; *p* = 0.30; I^2^ = 66%). At >12 months (*n* = 2 studies), the pooled OR was 1.81 (95% CI 0.90–3.64; *p* = 0.10; I^2^ = 48%). See Figs 5–7.

**Fig 8:**
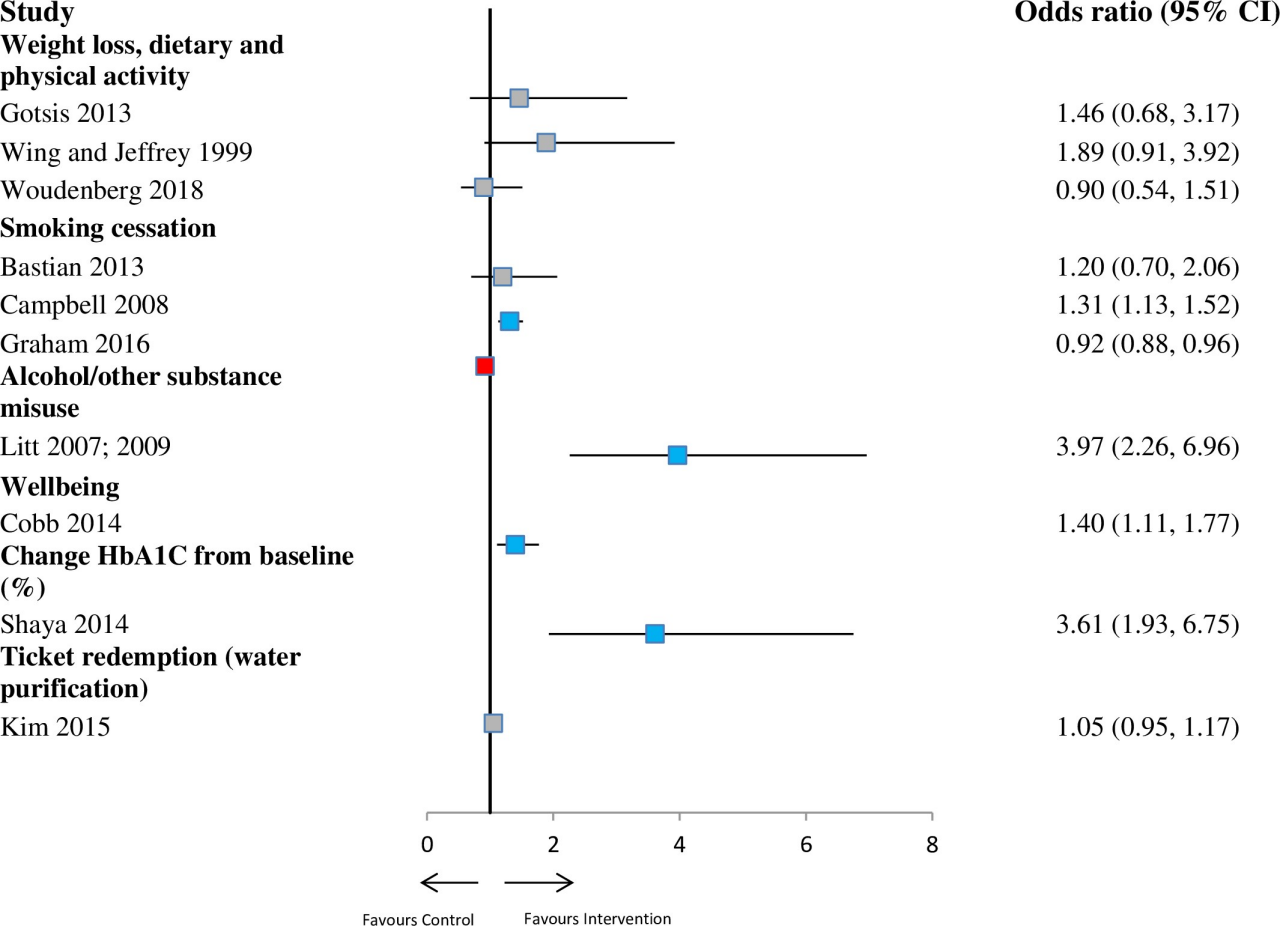
Forest plot showing odds for intervention groups compared with controls for outcomes reported at ≤6 months (other outcome measures). The blue denotes significant effect in favour of the Intervention group. The grey denotes non-significant. The red denotes significant effect in the favour of the control group. CI, confidence interval, HbA1c, haemoglobin A1c.

Effect size calculations from single studies showed a significant intervention effect for other outcomes including alcohol misuse at ≤6 months (OR 3.97; 95% CI 2.26–6.96), 6–12 months (OR 2.90; 95% CI 1.66–5.06), and >12 months (OR 1.77; 95% CI 1.00–3.13). There was a significant intervention effect at ≤6 months for change in well-being (OR 1.40; 95% CI 1.11–1.77) and HbA1c (OR 3.61; 95% CI 1.93–6.75). There was also a significant intervention effect for smoking cessation in adolescents at ≤6 months (OR 1.31; 95% CI 1.13–1.52), 6–12 months (OR 1.25; 95% CI 1.11–1.40), and >12 months (OR 1.19; 95% CI 1.08–1.31). See Fig 8 and Table 1. No significant intervention effects were found for several outcomes of single studies, including physical activity, diet, weight loss, and screening.

The strongest evidence for network intervention approach was for the individuals approach. Subgroup analyses provided evidence to support the individuals intervention approach for sexual health (S2–S4 Figs) (<6 months: 2.09 [1.63, 2.67] and 6–12 months: 1.62 [1.35, 1.95]) and drug risk (S5–S7 Figs) (<6 months: 4.68 [2.20, 9.96]). The results of all other subgroup analyses were not significant. Further details regarding the evidence for specific network intervention approaches are provided in the S3 Text.

For sexual health outcomes, other subgroup analyses were significant for intervention length (*p* > 0.05; S8 Fig) at ≤6 months, mean age of participants at ≤6 months (*p* = 0.002; S14 Fig), and percentage of female participants at >12 months (*p* < 0.001; S22 Fig). The results of all other subgroup analyses were not significant.

For sexual health outcomes reported at ≤6 months, sensitivity analyses showed that there was a significantly higher pooled OR for studies with an attrition rate of below 20% versus studies with a higher attrition rate (*p* = 0.002; S38 Fig). For sexual health outcomes reported at ≤6 months, sensitivity analyses also showed that there was a significantly higher pooled OR for studies that used RCT or cluster RCT (cRCT) designs versus studies that used any other design (*p* = 0.002; S44 Fig). For sexual health outcomes reported at >6 months to <12 months or less, sensitivity analyses showed that there was a significantly higher pooled OR for studies that used RCT or cRCT designs versus studies that used any other design (*p* = 0.003; S45 Fig). For drug risk outcomes reported at ≤6 months, sensitivity analyses showed that there was a significantly lower pooled OR for studies that used RCT or cRCT designs versus studies that used any other design (*p* < 0.001; S47 Fig). For all other analyses, sensitivity analyses showed that the pooled effect size estimate was robust to the omission of studies classified at high risk of bias, studies not conducting an ITT analysis, studies with high attrition rates, and studies using nonrandomised designs (see S26–S48 Figs).

Visual inspection of funnel plots revealed little evidence of publication bias for the examined studies (see S50–S53 Figs). Both the Egger and PEESE methods showed no evidence of small-study effects (*p* > 0.05) at all time points for sexual health and drug risk outcomes. However, the results of these tests, and of the meta-analyses themselves, should be interpreted cautiously because of the high degree of heterogeneity.

Overall, 15 studies had low risk of bias, and 22 had high risk of bias, leading to an overall high risk of bias across the included studies. The most common problem areas were inadequate randomisation methods and a lack of control for contamination between groups. Details of the risk of bias assessment for all included studies are available in the S1 Fig.

## Discussion

Findings from our review offer evidence of the effectiveness of social network interventions for health behaviours and outcomes. There is evidence to support both short-term (<6 months) and longer-term effects (>6 months), particularly for sexual health outcomes. Interventions using social network approaches support the repeated calls for health behaviour interventions to move beyond individual-level behaviour approaches [22,23] to exploit network influences on behaviour that have been well documented in the literature. The strongest evidence of effectiveness for a network intervention approach was for the individuals approach. Subgroup analyses provided evidence to support this individual approach for sexual health (<6 months: 2.09 [1.63, 2.67] and 6–12 months: 1.62 [1.35, 1.95]) and drug risk (<6 months: 4.68 [2.20, 9.96]).

Encouragingly, social network interventions have been successful in reaching, retaining, and changing the behaviour of so-called hidden, hard-to-reach, and at-risk populations, including MSM, people who inject drugs (PIDs), and other priority populations (e.g., low-income or minority populations). Overall, the included studies demonstrated high participation and retention rates that are critical for these interventions, which aim to activate and stimulate network and social environment mechanisms that promote health. Theoretically, such interventions work by redefining social norms towards the avoidance of high-risk behaviours. Typically, social norms are formed by observing the behaviour of popular peers and then adopting and modelling these behaviours, which further spreads them to others in the network [30].

The network interventions reviewed used or promoted existing network functions such as social identity, social support, social exchange, and social learning processes. Research has shown support for the social contagion theory [31], which suggests that health outcomes, behaviours, and beliefs (e.g., obesity, happiness) are ‘transmitted’ through social networks [32]. The employment of network theories and social–ecological models could build on the wealth of knowledge we have from individual-level models and their articulation of how social factors influence individual biology, beliefs, decisions, and behaviours. Furthermore, health behaviour interventions have traditionally been unsuccessful in achieving long-term maintenance of new behaviours; however, the important role of social networks in behaviour maintenance is evident in theories of habit formation (which emphasise the importance of external cues) [33] and theories of maintained behaviour change (which emphasise the role of social norms in reinforcing new behaviours) [34].

Notwithstanding the fact that the reviewed studies were of variable quality, fundamental methodological quality issues actually run much deeper, for the correct way to obtain unbiased effect estimates is not obvious and certainly nontrivial when individual observations fail the independence assumptions that are required for conventional analysis. Epidemiologists have only recently developed innovative methods to obtain valid estimates of putative causal effects, and the conceptual issues concerning control of confounding become somewhat more subtle in the presence of spill-over effects and interference [35]. Valente’s taxonomy of network intervention types was not specifically designed to correspond with putative mechanisms, as might be extracted from modern causal mediation analysis [36]. Network interventions have also been criticised for lacking generalisability and being context specific, given that network structures and dynamics are often shaped by local settings and environments. Entirely new evaluation designs may be required, and the suggestions made so far involving sequential randomisation have not been practical [37].

If social network interventions are to meaningfully inform public health policy and practice, then a number of implementation factors must be overcome in order to develop simple, cost-effective programmes. For example, innovative ‘network-optimised interventions’ are able to identify structurally influential individuals without mapping entire networks [13].

One limitation of our review is the difficulty of isolating the effects of specific social network intervention components on health outcomes. The social network interventions included in this review may have included other active intervention components, and the studies were not typically designed to test causal effects of social network mechanisms or to differentiate the effects of the four types of network intervention approaches. As Tanner-Smith and Grant have suggested, network science itself may offer added value in synthesising the evidence for these different types of network interventions [38]. Further studies are needed to rigorously evaluate different group-segmentation and leader-identification techniques and individuals network approaches such as identifying peripheral nodes, bridging nodes, and early adopters. The development of such interventions would benefit from the application of a multiphase optimization strategy (MOST) [39], which uses a factorial experimental design to identify the unique effects, and interaction effects, of specific intervention components that could include social network approaches or other active components (e.g., educational material). This would help to inform the optimal design for assessing the effectiveness and cost-effectiveness of network interventions. Further limitations include the reliance on self-reported outcomes, which have inherent recall and desirability biases, and results obtained in studies of key populations may limit the generalisability of findings to other populations or groups.

The cost-effectiveness of network-based interventions has yet to be established. We hypothesise that such interventions would be cost-effective given their reliance on ‘free’ human capital and potential to increase the reach, effectiveness, and maintenance of health behaviour interventions [40]. To the best of our knowledge, only one study conducted a cost-effectiveness analysis [41], and it found the intervention to be cost-effective and is now implemented at scale using a social enterprise model [42]. Kim and colleagues (2015) [13] suggested that network interventions could be particularly useful in resource- and infrastructure-limited areas, where networks could be used to increase the number of people engaged or could enhance the spread and adoption of those interventions.

An additional priority for future research should be adolescent populations. The strong empirical evidence base for the association between social networks and health behaviours in adolescents has rarely been applied in interventions in this population, and we found only two studies among adolescent populations. Therefore, given the compelling evidence base, including strong evidence for peer influence on adolescent health behaviours, and rich information on social network mechanisms in well-defined social network structures (typically peer networks within school settings), this area is ripe for network-based interventions, which would bridge the gap between the empirical evidence and current intervention approaches. Furthermore, none of the included studies involved social networking platforms. The pervasive use of online social networking platforms, particularly in adolescents, may help drive evaluative and methodological innovation for network interventions. Social networking sites inherently have extant social networks and should be further explored, albeit in light of acknowledged ethical and implementation challenges [43]. These platforms may hold promise for diffusion of information and ‘simple’ behaviour change [44], although initial evidence suggests online social ties may have less impact on complex behaviour change [45].

In summary, our systematic review and meta-analysis was reported in line with PRISMA, following a registered protocol and assessing risk of bias using a well-established tool to provide what we believe to be the first systematic review and meta-analysis of the effectiveness of social network interventions for a range of health outcomes. The evidence demonstrates that health and well-being are connected through complex and dynamic webs of social networks and that harnessing these networks may be especially important for health behaviour. However, existing health interventions rarely include components designed to explicitly use social network phenomena to maximise the adoption or diffusion of health-related information or behaviour. As evident from our review, social network interventions have demonstrated evidence of intervention effectiveness both in the short and long term, across a range of behaviours, settings, and populations. We argue that network phenomena are inherent in our interventions but are currently being overlooked and subsequently underused [5]. In light of these findings, future research and health behaviour interventions should account for the social networks in which individuals are embedded.

## Supporting information

S1 PRISMA Checklist (DOC)

S1 Text Methodology—Details of methods and search strategies. (DOCX)

S2 Text References of included studies. (DOCX)

S3 Text Subgroup analyses and forest plots. (DOCX)

S4 Text Sensitivity analyses. (DOCX)

S5 Text Publication bias. (DOCX)

S1 Table Social network functions for individual network interventions. (DOCX)

S2 Table Social network functions for segmentation network interventions. (DOCX)

S3 Table Social network functions for induction network interventions. (DOCX)

S4 Table Social network functions for alteration network interventions. (DOCX)

S1 Fig Risk of bias of included studies. (DOCX)

S2 Fig Forest plot for subgroup analysis of sexual health outcomes reported at ≤6 months: Intervention approach (individual, segmentation, induction, alteration). (DOCX)

S3 Fig Forest plot for subgroup analysis of sexual health outcomes reported at >6 months to <12 months: Intervention approach (individual, segmentation, induction, alteration). (DOCX)

S4 Fig Forest plot for subgroup analysis of sexual health outcomes reported at last follow-up: Intervention approach (individual, segmentation, induction, alteration). (DOCX)

S5 Fig Forest plot for subgroup analysis of drug risk outcomes reported at ≤6 months: Intervention approach (individual, segmentation, induction, alteration). (DOCX)

S6 Fig Forest plot for subgroup analysis of drug risk outcomes reported at >6 months to <12 months: Intervention approach (individual, segmentation, induction, alteration). (DOCX)

S7 Fig Forest plot for subgroup analysis of drug risk outcomes reported at last follow-up: Intervention approach (individual, segmentation, induction, alteration). (DOCX)

S8 Fig Forest plot for subgroup analysis of sexual health outcomes reported at ≤6 months: Intervention length (3 months or less; 3–6 months or less; 6 months or longer). (DOCX)

S9 Fig Forest plot for subgroup analysis of sexual health outcomes reported at >6 months to <12 months: Intervention length (3 months or less; 3–6 months or less; 6–12 months or less; 12–18 months or less; greater than 18 months). (DOCX)

S10 Fig Forest plot for subgroup analysis of sexual health outcomes reported at last follow-up: Intervention length (3 months or less; 3–6 months or less; 6–12 months or less; 12–18 months or less; greater than 18 months). (DOCX)

S11 Fig Forest plot for subgroup analysis of drug risk outcomes reported at ≤6 months: Intervention length (3 months or less; 3–6 months or less; 6 months or longer). (DOCX)

S12 Fig Forest plot for subgroup analysis of drug risk outcomes reported at >6 months to <12 months: Intervention length (3 months or less; 3–6 months or less; 6–12 months or less; 12–18 months or less; greater than 18 months). (DOCX)

S13 Fig Forest plot for subgroup analysis of drug risk outcomes reported at last follow-up: Intervention length (3 months or less; 3–6 months or less; 6–12 months or less; 12–18 months or less; greater than 18 months). (DOCX)

S14 Fig Forest plot for subgroup analysis of sexual health outcomes reported at ≤6 months: Participant age (above or below mean age of 32.4 years across studies). (DOCX)

S15 Fig Forest plot for subgroup analysis of sexual health outcomes reported at >6 months to <12 months: Participant age (above or below mean age of 32.4 years across studies). (DOCX)

S16 Fig Forest plot for subgroup analysis of sexual health outcomes reported at last follow-up: Participant age (above or below mean age of 32.4 years across studies). (DOCX)

S17 Fig Forest plot for subgroup analysis of drug risk outcomes reported at ≤6 months: Participant age (above or below mean age of 32.4 years across studies). (DOCX)

S18 Fig Forest plot for subgroup analysis of drug risk outcomes reported at >6 months to <12 months: Participant age (above or below mean age of 32.4 years across studies). (DOCX)

S19 Fig Forest plot for subgroup analysis of drug risk outcomes reported at last follow-up: Participant age (above or below mean age of 32.4 years across studies). (DOCX)

S20 Fig Forest plot for subgroup analysis of sexual health outcomes reported at ≤6 months: Participant gender (above or below mean of 45.5% female across studies). (DOCX)

S21 Fig Forest plot for subgroup analysis of sexual health outcomes reported at >6 months to <12 months: Participant gender (above or below mean of 45.5% female across studies). (DOCX)

S22 Fig Forest plot for subgroup analysis of sexual health outcomes reported at last follow-up: participant gender (above or below mean of 45.5% female across studies). (DOCX)

S23 Fig Forest plot for subgroup analysis of drug risk outcomes reported at ≤6 months: Participant gender (above or below mean of 45.5% female across studies). (DOCX)

S24 Fig Forest plot for subgroup analysis of drug risk outcomes reported at >6 months to <12 months: Participant gender (above or below mean of 45.5% female across studies). (DOCX)

S25 Fig Forest plot for subgroup analysis of drug risk outcomes reported at last follow-up: Participant gender (above or below mean of 45.5% female across studies). (DOCX)

S26 Fig Forest plot for sensitivity analysis of sexual health outcomes reported at ≤6 months: Risk of bias. (DOCX)

S27 Fig Forest plot for sensitivity analysis of sexual health outcomes reported at >6 months to <12 months: Risk of bias. (DOCX)

S28 Fig Forest plot for sensitivity analysis of sexual health outcomes reported at last follow-up: Risk of bias. (DOCX)

S29 Fig Forest plot for sensitivity analysis of drug risk outcomes reported at ≤6 months: Risk of bias. (DOCX)

S30 Fig Forest plot for sensitivity analysis of drug risk outcomes reported at >6 months to <12 months: Risk of bias. (DOCX)

S31 Fig Forest plot for sensitivity analysis of drug risk outcomes reported at last follow-up: Risk of bias. (DOCX)

S32 Fig Forest plot for sensitivity analysis of sexual health outcomes reported at ≤6 months: Intention-to-treat analysis. (DOCX)

S33 Fig Forest plot for sensitivity analysis of sexual health outcomes reported at >6 months to <12 months: Intention-to-treat analysis. (DOCX)

S34 Fig Forest plot for sensitivity analysis of sexual health outcomes reported at last follow-up: Intention-to-treat. (DOCX)

S35 Fig Forest plot for sensitivity analysis of drug risk outcomes reported at ≤6 months: Intention-to-treat analysis. (DOCX)

S36 Fig Forest plot for sensitivity analysis of drug risk outcomes reported at >6 months to <12 months: Intention-to-treat analysis. (DOCX)

S37 Fig Forest plot for sensitivity analysis of drug risk outcomes reported at last follow-up: Intention-to-treat. (DOCX)

S38 Fig Forest plot for sensitivity analysis of sexual health outcomes reported at ≤6 months: Attrition rate. (DOCX)

S39 Fig Forest plot for sensitivity analysis of sexual health outcomes reported at >6 months to <12 months: Attrition rate. (DOCX)

S40 Fig Forest plot for sensitivity analysis of sexual health outcomes reported at last follow-up: Attrition rate. (DOCX)

S41 Fig Forest plot for sensitivity analysis of drug risk outcomes reported at ≤6 months: Attrition rate. (DOCX)

S42 Fig Forest plot for sensitivity analysis of drug risk outcomes reported at >6 months to <12 months: Attrition rate. (DOCX)

S43 Fig Forest plot for sensitivity analysis of drug risk outcomes reported at last follow-up: Attrition rate. (DOCX)

S44 Fig Forest plot for sensitivity analysis of sexual health outcomes reported at ≤6 months: Study design. (DOCX)

S45 Fig Forest plot for sensitivity analysis of sexual health outcomes reported at >6 months to <12 months: Study design. (DOCX)

S46 Fig Forest plot for sensitivity analysis of sexual health outcomes reported at last follow-up: Study design. (DOCX)

S47 Fig Forest plot for sensitivity analysis of drug risk outcomes reported at ≤6 months: Study design. (DOCX)

S48 Fig Forest plot for sensitivity analysis of drug risk outcomes reported at >6 months to <12 months: Study design. (DOCX)

S49 Fig Forest plot for sensitivity analysis of drug risk outcomes reported at last follow-up: Study design. (DOCX)

S50 Fig Funnel plot for sexual health outcome measures reported at ≤6 months. (DOCX)

S51 Fig Funnel plot for sexual health outcome measures reported at >6 months to <12 months. (DOCX)

S52 Fig Funnel plot for sexual health outcome measures reported at last follow-up. (DOCX)

S53 Fig Funnel plot for drug risk outcome measures reported at ≤6 months. (DOCX)

## Abbreviations

CENTRAL: Cochrane Central Register of Controlled Trials
CI: confidence interval
cRCT: cluster RCT
DAC: Development Assistance Committee
ERIC: Education Resources Information Center
HbA1c: haemoglobin A1c
HIV: human immunodeficiency virus
IBSS: International Bibliography of the Social Sciences
ICTRP: International Clinical Trials Registry Portal
ITT: intention-to-treat
LMIC: low- and middle-income country
MOST: multiphase optimization strategy
MSM: men who have sex with men
ODA: Official Development Assistance
OR: odds ratio
PEESE: precision-effect estimate with standard error
PID: person who injects drugs
PRISMA: Preferred Reporting Items for Systematic Reviews and Meta-Analyses
RCT: randomised controlled trial
SE: standard error
SMD: standardised mean difference
UAI: unprotected anal intercourse
UI: unprotected intercourse
UVI: unprotected vaginal intercourse
WHO: World Health Organization
