# Supplementary material for: Social network interventions for health behaviours and outcomes: A systematic review and meta-analysis
Source: PLoS Med. 2019 Sep 3;16(9):e1002890. doi: 10.1371/journal.pmed.1002890 (PMC6719831; doi:10.1371/journal.pmed.1002890)
Supplement: S1 Text — (DOCX) [file pmed.1002890.s002.docx]

**S1 Text: Methodology-Details of methods and search strategies**

The following provides further details regarding the interventions included in the review. Interventions were categorised according to the specific network approach. A taxonomy of social network intervention approaches was established in 2012 [1]. Four intervention approaches are described in detail below.

1. Individuals Network Approaches

Individual network interventions included those which specifically used network data to identify certain individuals to be recruited to act as proponents of behaviour change on the basis of some network property. For example, peer nominations used by members of the social network to identify leaders to promote behaviour change. Different mathematical algorithms are available to analyse social network data in order to identify individuals specific social positions in a network.

Examples include:

- Centrality degree: identifies the individuals with the most ties;
- Centrality closeness: identifies individuals that can reach everyone else in fewer steps;
- Centrality betweenness: identifies individuals that occupy critical gate-keeping positions by most frequently lying on the shortest path connecting other nodes;
- Key players: identifies individuals based on centrality and network communities who would be the most relevant seeds for spreading a behaviour;
- Bridging: identifies individuals in “brokering” positions who have many connections to people who are not directly connected to each other, or who play a bridging role in that their connections maximally increase network cohesion;
- Low-threshold nodes: identifies individuals who are early adopters of the behaviour;
- Peripheral nodes: identifies individuals on the margins of the network who have ties to individuals with low centrality.

1. Segmentation Network Approaches

Segmentation network interventions included interventions directed towards groups of people clustered in a network. In network science, clusters refer to groups with a relatively high density of connections between them. Clusters of people within a network are encouraged to change their behaviour at the same time, with the group sessions being facilitated by a non-network member, mainly by trained facilitators.

1. Induction Network Approaches

Induction network interventions involve excitation and activation of existing social ties in a social network to diffuse information or healthy behaviours. Stimulation of these peer-to-peer interactions can occur via a number of mechanisms such as i) Word-of mouth (WOM) interventions which stimulate interpersonal communication and/or support to increase the probability that others will adopt the new behaviour; ii) respondent-driven (RDS) or snowball sampling in which individuals recruit others to participate in a study or receive an intervention; iii) Network outreach which is similar to RDS, except that the network seeds recruit members of their personal networks to participate in an intervention together, in which the behaviour change messages can be delivered to the entire group.

1. Alteration Network Approaches

Alteration network interventions involve changing the structure of the network by the addition of new members or breaking existing ties with those who foster and facilitate unhealthy, risky behaviours. Three main strategies include: (i) adding and/or deleting nodes, (ii) adding and/or deleting ties, or (iii) rewiring existing ties in the network.

***Search strategy***

The following search terms were combined together along with truncation appropriate to individual databases.

1. exp tobacco/

2. exp "Tobacco Use Cessation"/

3. exp “drinking behavior”/

4. exp “alcohol drinking”/

5. exp food habits/

6. exp diet/

7. exp exercise/

8. weight loss/

9. immunization/

10. mass screening/

11. substance-related disorders/

12. safe sex/

13. health behavior/

14. health promotion/

15. 1 or 2 or 3 or 4 or 5 or 6 or 7 or 8 or 9 or 10 or 11 or 12 or 13 or 14

16. alcohol.ti,ab.

17. "cigarette$". ti,ab.

18. smoking. ti,ab.

19. tobacco. ti,ab.

20. (diet adj behavio$). ti,ab.

21. (healthy adj diet). ti,ab.

22. (healthy adj food). ti,ab.

23. (healthy adj habit). ti,ab.

24. (healthy adj eating). ti,ab.

25. "nutrition$". ti,ab.

26. (physical* adj active*). ti,ab.

27. (exercise or exerc* or cycl* or walk* or bik* or bicycle* or recreation* or leisure* or sport*). ti,ab.

28. drinking. ti,ab.

29. (immunisation or immunization). ti,ab.

30. (immunise or immunize). ti,ab.

31. screening. ti,ab.

32. "vaccinat$". ti,ab.

33. (weight adj loss). ti,ab.

34. (weight adj maintenance). ti,ab.

35. (weight adj management). ti,ab.

36. (weight adj reduction). ti,ab

37. (safe adj sex). ti,ab.

38. (sexual adj health). ti,ab.

39. (Substance adj abuse). ti,ab

40. (Drug adj abuse). ti,ab

41. 16 or 17 or 18 or 19 or 20 or 21 or 22 or 23 or 24 or 25 or 26 or 27 or 28 or 29 or 30 or 31 or 32 or 33 or 34 or 35 or 36 or 37 or 38 or 39 or 40

42. exp social support/

43. (social adj2 network).tw

44. (network OR networks).ti,ab

45. (Network social).tw

46. (social adj2 network adj2 intervention).tw

47. 42 or 43 or 44 or 45 or 46

48. 15 OR 41

50. 47 AND 48

51. 50 limited to English language, humans, published in 1990 and onwards.

Abbreviations: mp=keywords; “/”=MeSh headings; ti,ab-title and abstract; adj=adjacent; exp=explode; tw=text words; $ and * denote truncation

***Screening***

Reference lists of included studies were also screened for eligible studies. Any disagreements were resolved by discussion between members of the research team. Independent screeners exhibited high reliability and agreement for the screening of titles and abstracts (κ=0.66, agreement=82%), full-texts (κ =0.68, agreement=87%) [2].

***Data extraction***

Where publications lacked sufficient detail for full data extraction, we contacted the original authors for the necessary information. Authors of six included studies were contacted via email with requests for data, or clarification of results reported in articles; four replied with the requested information or stated that it was unavailable.

The umbrella term “Social Network Functions” was used to describe the core elements of the intervention approaches, including the network intervention strategy employed and the underpinning theoretical mechanisms, definition and boundary of the social network, network recruitment methods (where appropriate), training methods, and any details regarding the structure and characteristics of the social network, or change in the social network described using network parameters.

***Data synthesis and statistical analysis***

The potential for meta-analyses was explored for all outcomes. Meta-analyses were conducted according to time-point of outcome measurement (≤6 months, six-≤12 months, last follow-up) for sexual health outcomes and drug risk outcomes. However, due to the heterogeneity in target populations, health behaviours and outcome measures, it was not appropriate to pool data in a meta-analysis for other outcomes.

*Outcome measures and effect size calculation*

Meta-analysis included a range of objective and self-report measures of percentage of participants engaging in condomless sex, percentage of participants engaging in injection drug risk or other drug risk behaviours, percentage of participants becoming HIV-positive or contracting another sexually transmitted infection (STI) from baseline. Other outcomes included total fruit and vegetable consumption, weight loss from baseline, change in HbA1C from baseline, proportion of days abstinent from alcohol consumption, smoking cessation, percentage of participants engaging in mammography use, proportion of tickets redeemed for water purification purposes, rates of intervention engagement, participant wellbeing, days/week engaged in physical activity (PA) and percentage of participants engaging in contraception use.

Meta-analyses were conducted according to timepoint of outcome measurement (≤six months, six-≤12 months, last follow-up). Log odds ratios (ORs) and standard errors (SEs) were calculated for each study representing the odds of achieving a more favourable outcome for an intervention group compared to controls. Where studies reported event (numbers or percentages of participants) and total data (*k*=21) or adjusted ORs and 95% confidence intervals (CIs) (*k*=6), these were used to directly compute log ORs and SEs. When studies reported ORs and p-values, p-values were converted to SEs using procedures outlined in the Cochrane handbook [3] (*k*=1). When studies reported means and standard deviations (SDs), standardised mean differences (SMDs) and SEs were calculated and converted to log ORs and SEs using the Chinn (2000) equation [4] (*k*=12). When studies reported data separately for multiple intervention groups or subgroups, data were combined using procedures outlined in the Cochrane handbook [3] (*k*=5). All data were transformed so that higher OR values indicated higher odds of achieving a more favourable outcome for intervention groups compared to controls. A significant intervention effect was determined when the 95% CI excluded 1 for the OR. Separate meta-analyses were carried out for studies reporting: (1) sexual health outcomes; (2) drug risk outcomes.

Cochran’s *Q* was calculated and Chi-square tests determined whether variation observed between study results was consistent with sampling error only. Cochran’s *Q* was transformed to I-squared statistics (i.e. the percentage of variability in ORs due to heterogeneity between studies rather than chance) [3]. Heterogeneity and reporting bias were assessed visually using forest and funnel plots created using Review Manager [5]. The Egger et al. (1997) [6] and precision-effect estimate with standard error (PEESE) [7] tests for study size effects were used to formally test for publication bias. This was carried out by performing weighted least squares regressions with log OR effect sizes as the dependent variable. The independent variable for the Egger test was the log OR standard error (SE) whilst the squared standard error (SE^2^) was used as the independent variable for the PEESE test. Regression weights were specified as 1/ SE^2^. The intercept was interpreted as the mean log OR effect size adjusted for publication bias. Statistical significance was set at the 5% level (p<0.05).

*Sensitivity analyses*

Sensitivity analyses were conducted to determine whether the results of the meta-analysis were robust to omission of studies classified at high risk of bias using the Cochrane Risk of Bias tool, studies not performing Intention-to-treat (ITT) analyses, studies with attrition rates higher than 20%, and studies using non-randomised designs. The literature suggests that attrition rates >20% should raise concern about the possibility of bias [8] and use of ITT analysis is recommended to minimise bias [3,9].

*Risk of bias*

Risk of bias was assessed using the Cochrane Risk of Bias tool [3], and categorised as ‘high’, ‘low’ or ‘unclear’ for each domain. Domains included:

- **Allocation sequence:** Was the allocation sequence adequately generated?
- **Allocation concealment:** Was allocation adequately concealed?
- **Baseline measurements:** Were baseline outcome measurements similar?
- **Baseline characteristics**: Were baseline characteristics similar?
- **Incomplete data:** Were incomplete outcome data adequately addressed?
- **Blinding:** Was knowledge of the allocated intervention adequately prevented during the study?
- **Contamination:** Was the study adequately protected against contamination?
- **Selective reporting:** Are reports of the study free of suggestion of selective outcome reporting?

Two review authors independently evaluated the included studies for risk of bias according to the domains recommended by Cochrane [3] outlined above. Where information was not included in the manuscript, we labelled risk of bias as unclear.

*Allocation sequence:* refers to methods used to randomise people to study groups. Adequate random sequence generation that were considered to be low risk of bias included use of a random number generator or random number table.

*Allocation concealment:* methods that prevent study team members from knowing the group assignment of study participants. We assigned low risk of bias where adequate allocation concealment methods such as central allocation or the use of sequentially numbered opaque, sealed envelopes that contain study assignments were reported.

*Baseline measurements:* Studies were classified as low risk of bias if a standardised measurement protocol was followed for baseline outcome measurements in all groups.

*Baseline characteristics:* Studies were classified as low risk of bias if baseline characteristics (particularly for key sociodemographic characteristics and primary outcome(s)) were similar or appropriate statistical methods were employed to account for any baseline differences.

*Incomplete outcome data:* refers to differential attrition between groups and appropriate handling of missing data. Studies were classified as low risk of bias if they had non‐differential attrition between groups and used intention‐to‐treat principles.

*Blinding (of participants, study team members and outcome assessors):* refers to methods to prevent the participants, study team members (i.e. those who delivered the intervention) and outcome assessors (i.e. those involved in data collection and/or data analysis) from being aware of the participant's group allocation. We classified studies as low risk of bias if the participants, study team members and outcome assessors were blinded to study group allocation.

*Contamination:* Studies were classified as low risk of bias if appropriate methods were reported to ensure that participants in different groups did not meet.

*Selective reporting:* refers to consistency in the outcomes reported in the methods or study protocol (if available) and those reported in the results. Studies were classified as low risk of bias if a protocol or prior publication was available whereby outcomes were clearly designated a priori and reported in the included study.

**References**

1. Valente TW. Network interventions. Science 2012;337: 49—53.

2. Landis JR, Koch GG. The measurement of observer agreement for categorical data. Biometrics 1977;33: 159—174.

3. Higgins JP, Green S. Cochrane Handbook for Systematic Reviews of Interventions version 5.1.0 [updated March 2011]. Retrieved from http://handbook.cochrane.org/

4. Chinn S. A simple method for converting an odds ratio to effect size for use in meta-analysis. Stat Med 2000;19: 3127—3131.

5. The Cochrane Collaboration. (2014). Review Manager (RevMan) [Computer program]. Version 5.3. Copenhagen: The Nordic Cochrane Centre, The Cochrane Collaboration.

6. Egger M, Davey Smith G, Schneider M, Minder C. Bias in meta-analysis detected by a simple, graphical test. BMJ 1997;315: 629—634.

7. Stanley TD, Doucouliagos H. Meta-regression approximations to reduce publication selection bias. Res Synth Methods 2014;5: 60—78.

8. Schulz KF, Grimes DA. Sample size slippages in randomised trials: exclusions and the lost and wayward. Lancet 2002; 359: 781—785.

9. Newell DJ. Intention-to-treat analysis: implications for quantitative and qualitative research. Int J Epidemiol 1992;21: 837—841.
