# Supplementary material for: Social network interventions for health behaviours and outcomes: A systematic review and meta-analysis
Source: PLoS Med. 2019 Sep 3;16(9):e1002890. doi: 10.1371/journal.pmed.1002890 (PMC6719831; doi:10.1371/journal.pmed.1002890)
Supplement: S2 Text — (DOCX) [file pmed.1002890.s003.docx]

**S2 Text: References of included studies**

1. Kelly JA, Murphy DA, Sikkema KJ, McAuliffe TL, Roffman RA, Solomon LJ, et al. Randomised, controlled, community-level HIV-prevention intervention for sexual-risk behaviour among homosexual men in US cities. Community HIV Prevention Research Collaborative. Lancet. 1997;350: 1500—1505.

2. Latkin CA. Outreach in natural settings: the use of peer leaders for HIV prevention among injecting drug users' networks. Public Health Rep. 1998;113: 151—159.

3. Sikkema KJ, Kelly JA, Winett RA, Solomon LJ, Cargill VA, Roffman RA, et al. Outcomes of a randomized community-level HIV prevention intervention for women living in 18 low-income housing developments. Am J Public Health. 2000;90: 57—63.

4. Amirkhanian YA, Kelly JA, Kabakchieva E, Kirsanova AV, Vassileva S, Takacs J, et al. A randomized social network HIV prevention trial with young men who have sex with men in Russia and Bulgaria. AIDS. 2005;19: 1897—1905.

5. Kelly JA, Amirkhanian YA, Kabakchieva E, Vassileva S, Vassilev B, McAuliffe TL, et al. Prevention of HIV and sexually transmitted diseases in high risk social networks of young Roma (Gypsy) men in Bulgaria: randomised controlled trial. BMJ. 2006;333: 1098.

6. Campbell R, Starkey F, Holliday J, Audrey S, Bloor M, Parry-Langdon N, et al. An informal school-based peer-led intervention for smoking prevention in adolescence (ASSIST): a cluster randomised trial. Lancet 2008;371: 1595—1602.

7. Kim DA, Hwong AR, Stafford D, Hughes DA, O'Malley AJ, Fowler JH, et al. Social network targeting to maximise population behaviour change: a cluster randomised controlled trial. Lancet. 2015;386: 145-153. doi: 10.1016/S0140-6736(15)60095-2.

8. Amirkhanian YA, Kelly JA, Takacs J, McAuliffe TL, Kuznetsova AV, Toth TP, et al. Effects of a social network HIV/STD prevention intervention for MSM in Russia and Hungary: a randomized controlled trial. AIDS. 2015; 29: 583—593. doi: 10.1097/QAD.0000000000000558.

9. van Woudenberg TJ, Bevelander KE, Burk WJ, Smit CR, Buijs L, Buijzen M. A randomized controlled trial testing a social network intervention to promote physical activity among adolescents. BMC Public Health. 2018;18: 542. doi: 10.1186/s12889-018-5451-4.

10. Trotter RT, Bowen AM. The efficacy of network-based HIV/AIDS risk reduction programs in mid-sized towns in the United States. J Drug Issues. 1996; 26 (3).

11. Kincaid DL. Social networks, ideation, and contraceptive behavior in Bangladesh: a longitudinal analysis. Soc Sci Med. 2000;50: 215—231.

12. Minnis AM, van Dommelen-Gonzalez E, Luecke E, Dow W, Bautista-Arredondo S, Padian NS. Yo Puedo--a conditional cash transfer and life skills intervention to promote adolescent sexual health: results of a randomized feasibility study in san francisco. J Adolesc Health. 2014;55(1):85—92. doi: 10.1016/j.jadohealth.2013.12.007.

13. Shaya FT, Chirikov VV, Howard D, Foster C, Costas J, Snitker S, et al. Effect of social networks intervention in type 2 diabetes: a partial randomised study. J Epidemiol Community Health. 2014;68: 326—332. doi: 10.1136/jech-2013-203274.

14. Cobb NK, Josée PJ. Effectiveness of a multimodal online well-being intervention. A randomized controlled trial. Am J Prev Med. 2014;46: 41—48.

15. Kegeles SM, Hays RB, Coates TJ. The Mpowerment project: A community-level HIV prevention intervention for young gay men. Am J Public Health. 1996;86: 1129.

16. Latkin CA, Mandell W, Vlahov D, Oziemkowska M, Celentano DD. The long-term outcome of a personal network-oriented HIV prevention. Am J Community Psychol 1996;24: 341.

17. Buller DB, Morrill C, Taren D, Aickin M, Sennott-Miller L, Buller MK, et al. Randomized trial testing the effect of peer education at increasing fruit and vegetable intake. J Natl Cancer Inst. 1999;91: 1491—1500.

18. Wing RR, Jeffrey RW. Benefits of recruiting participants with friends and increasing social support for weight loss and maintenance. J Consult Clin Psychol 1999;67: 132-138.

19. Elford J, Bolding G, Sherr L. Peer education has no significant impact on HIV risk behaviours among gay men in London. AIDS. 2001;15: 535—538.

20. Earp JA, Eng E, O'Malley MS, Altpeter M, Rauscher G, Mayne L, et al. Increasing use of mammography among older, rural African American women: results from a community trial. Am J Public Health. 2002;92: 646—654.

21. Flowers P, Hart GJ, Williamson LM, Frankis JS, Der GJ. Does bar-based, peer-led sexual health promotion have a community-level effect amongst gay men in Scotland? Int J STD AIDS 2002;13: 102—108.

22. Latkin CA, Sherman S, Knowlton A. HIV prevention among drug users: outcome of a network-oriented peer outreach intervention. Health Psychol. 2003;22: 332—339.

23. Morisky DE, Ang A, Coly A, Tiglao TV. A model HIV/AIDS risk reduction programme in the Philippines: a comprehensive community-based approach through participatory action research. Health Promot Int. 2004;19: 69—76.

24. Garfein RS, Golub ET, Greenberg AE, Hagan H, Hanson DL, Hudson SM, et al. A peer-education intervention to reduce injection risk behaviors for HIV and hepatitis C virus infection in young injection drug users. AIDS. 2007;21: 1923—1932.

25. Valente TW, Ritt-Olson A, Stacy A, Unger JB, Okamoto J, Sussman S. Peer acceleration: effects of a social network tailored substance abuse prevention program among high-risk adolescents. Addiction. 2007;102: 1804—1815.

26. Latkin CA, Donnell D, Metzger D, Sherman S, Aramrattna A, Davis-Vogel A, et al. The efficacy of a network intervention to reduce HIV risk behaviors among drug users and risk partners in Chiang Mai, Thailand and Philadelphia, USA. Soc Sci Med. 2009;68: 740—748. doi: 10.1016/j.socscimed.2008.11.019.

27. Sutcliffe C, Srirojn B, Latkin CA, Aramratanna A, Sherman SG. Evaluation of a peer network intervention trial among young methamphetamine users in Chiang Mai, Thailand. Soc Sci Med. 2009;68: 69—79. doi: 10.1016/j.socscimed.2008.09.061.

28. Tobin KE, Kuramoto SJ, Davey-Rothwell MA, Latkin CA. The STEP into Action study: a peer-based, personal risk network-focused HIV prevention intervention with injection drug users in Baltimore, Maryland. Addiction. 2011;106: 366—375. doi: 10.1111/j.1360-0443.2010.03146.x.

29. Bastian LA, Fish LJ, Peterson BL, Biddle AK, Garst J, Lyna P, et al. Proactive recruitment of cancer patients' social networks into a smoking cessation trial. Contemp Clin Trials. 2011; 32: 498—504. doi: 10.1016/j.cct.2011.03.006.

30. Hoffman IF, Latkin CA, Kukhareva PV, Malov SV, Batluk JV, Shaboltas AV, et al. A peer-educator network HIV prevention intervention among injection drug users: results of a randomized controlled trial in St. Petersburg, Russia. AIDS Behav. 2013;17: 2510—2520. doi: 10.1007/s10461-013-0563-4.

31. Gotsis M, Wang H, Spruijt-Metz D, Jordan-Marsh M, Valente TW. Wellness partners: design and evaluation of a web-based physical activity diary with social gaming features for adults. JMIR Res Protoc. 2013;2: e10. doi: 10.2196/resprot.2132.

32. Booth RE, Davis JM, Dvoryak S, Brewster JT, Lisovska O, Strathdee SA, et al. HIV incidence among people who inject drugs (PWIDs) in Ukraine: results from a clustered randomised trial. Lancet HIV. 2016;3: e482-9. doi: 10.1016/S2352-3018(16)30040-6.

33. Cobb NK, Jacobs MA, Wileyto P, Valente T, Graham AL. Diffusion of an evidence-based smoking cessation intervention through Facebook: A randomized controlled trial. Am J Public Health. 2016;106: 1130—1135. doi: 10.2105/AJPH.2016.303106.

34. Wingood GM, DiClemente RJ, Mikhail I, Lang DL, Hubbard McRee D, et al. A randomized controlled trial to reduce HIV transmission risk behaviors and sexually transmitted diseases among women living with HIV. The WiLLOW program. J Acquir Immunc Defic Syndr. 2004; 37:S58—S67.

35. Litt MD, Kadden RM, Kabela-Cormier E, Petry N. Changing network support for drinking: initial findings from the network support project. J Consult Clin Psychol. 2007;75: 542—555.

36. Litt MD, Kadden RM, Kabela-Cormier E, Petry NM. Changing network support for drinking: network support project 2-year follow-up. J Consult Clin Psychol. 2009;77:229—242. doi: 10.1037/a0015252.

37. Eaton LA, Cherry C, Cain D, Pope H. A novel approach to prevention for at-risk HIV-negative men who have sex with men: creating a teachable moment to promote informed sexual decision-making. Am J Public Health. 2011;101:539—545. doi: 10.2105/AJPH.2010.191791.

38. Graham AL, Papandonatos GD, Cha S, Erar B, Amato MS, Cobb NK, Niaura RS, Abrams DB. Improving adherence to smoking cessation treatment: Intervention effects in a web-based randomized trial. Nicotine Tob Res. 2016. pii: ntw282.
