# Supplementary material for: Social network interventions for health behaviours and outcomes: A systematic review and meta-analysis
Source: PLoS Med. 2019 Sep 3;16(9):e1002890. doi: 10.1371/journal.pmed.1002890 (PMC6719831; doi:10.1371/journal.pmed.1002890)
Supplement: S3 Text — (DOCX) [file pmed.1002890.s004.docx]

**S3 Text: Subgroup analyses and forest plots**

***Intervention Approach (S2—S7 Fig)***

An objective of the sub-group analyses was to explore the evidence for network interventions approaches based on the Valente (2012) taxonomy. However, due to the clinical and methodological heterogeneity evidence in the included studies, sub-group analyses have been conducted for sexual health and drug risk outcomes only, where possible. This limited the number of comparisons and results from the sub-group analyses should be interpreted with caution due to the small numbers. As this was an important objective of the study we have therefore supplemented the sub-group analyses with a narrative synthesis of the evidence by intervention approach.

Individuals Approach: Sub-group analyses provided evidence to support the Individuals intervention approach for sexual health (S2—S4 Fig) (<6 months: 2.09 (1.63, 2.67) and 6—12 months: 1.62 (1.35, 1.95)) and drug risk (S5—S7 Fig) (<6 months: 4.68 (2.20, 9.96)). The majority of studies [1—7] employing an Individuals approach involved opinion or peer leaders identified as being the most popular or influential based on peer nominations pre-intervention, to act as peer educators or proponents of behaviour change. A different approach was taken by Amirkhanian and colleagues [4] who employed a novel HIV prevention intervention using socio-centric networks, who recruited three generations of MSM enabling the intervention to be delivered beyond small clusters of ego-networks to much larger complete socio-centric networks. Network members were more likely to receive HIV prevention messages from a number of ‘seeds’, thereby increasing the potential and support for behaviour change. Kim et al [7] tested different targeting approaches involving ‘in-degree’ (i.e. villagers named as a contact most often by others in their village), ‘nomination targeted’ (i.e. one randomly chosen friend nominated by each member of a 5% random sample of villagers) and ‘randomly targeted’ (i.e. targeted a random sample of villagers). Woudenberg et al [9] used peer nomination approaches in school classes to identify the most central participants based on closeness centrality to act as change agents in a physical activity intervention.

Segmentation Approach: Sub-group analyses provided evidence to support the Segmentation intervention approach for sexual health (at last follow-up: 7.88 (4.94, 12.57)). The Segmentation approach adopted by three interventions [10,12,13] involved ‘index participants’ (i.e. initial recruits), identified via community outreach or healthcare workers, who were asked to invite approximately 2—3 peers or network members to attend relevant education sessions with them, which were facilitated by someone outside of the network group (i.e. distinct from the opinion leaders approach where education sessions were facilitated by network members). Participants were encouraged to interact with network members to discuss prevention strategies taught in the group sessions. The intervention approach adopted by Kincaid (2000) [11] involved government family planning field workers holding meetings with village women in the homes of ‘opinion leaders’, defined as satisfied current adopters of contraceptive behaviour, who were centrally located from the network perspective, but geographically dispersed to cover the entire village network. The intervention utilised the existing informal social network of the village and provided a forum for the face-to-face discussion of family planning with other women in their village. Cobb et al (2014) [14] investigated a multimodal intervention (incorporating e-mail-, web-, and mobile) in which participants were asked to invite their ‘offline’ networks to a website to enhance wellbeing. Participants were encouraged and incentivised (e.g. awarded points, badges) to interact and establish new connections with other participants on the website, and form pacts to complete challenges together and encourage one another within a supportive social environment.

Induction approaches: Sub-group analyses provided non-significant evidence to support the use of Induction intervention approaches for drug risk (6—12 months: 1.40 (0.74, 2.64)). The majority of studies employed a network outreach approach [15—28,30,32]. Typically, these approaches involved seed participants recruiting members of their networks into the study or to receive the intervention. The behaviour change components of the intervention were then delivered to the entire group (clusters containing the seed and their participating networks) via a ‘leaders within group’ approach (e.g. Kegeles et al [15]) i.e. leaders within groups were identified and expected to induce behaviour change within their local networks or via a cohesive group approach (e.g. Tobin et al [28]). Typically, these studies involved respondent-driven sampling (RDS) where initial seeds were asked to recruit a number of others into the study to receive the intervention. One study used a snowballing recruitment approach which involved network members of initial seeds (1^st^ generation) to recruit members of their network (2^nd^ generation) and to become peer leaders themselves for their networks and deliver theme-based HIV prevention messages via daily social contacts (network outreach).

Six studies which employed a network outreach approach reported a non-significant intervention effect. Bastian et al (2013) [29] solely used a RDS recruitment approach where lung cancer patients were asked to recruit network members (family and close friends who were smokers) to a smoking quit intervention. However, it is important to note that a number of these studies experienced significant implementation barriers. For example, process evaluations by Elford et al [19] and Flowers et al [21] identified problems with recruiting and training a critical mass of opinion leaders, and a key limitation in the study by Hoffman et al [30] was that only a mean of 1.3 network participants per index participant were enrolled which is far short of the anticipated three per index participant required.

Alteration Approach: Education sessions facilitated by trained health workers and peer educators taught women with HIV ways of maintaining supportive network members, encouraged women to seek new network members, and informed participants how to disengage from unsupportive network members [34]. Results showed that the intervention group reported significantly fewer episodes of unprotected vaginal intercourse (UVI) (1.8 vs 2.5; p=0.022) compared with the control group. Results also showed a significant increase in the number of network members who provided social support in the intervention group (15.2 vs 13.6, p=0.02).

The intervention by Litt et al [35,36] demonstrated a significant intervention effect, which was also maintained at two years follow-up, for participants who misused alcohol. Results showed that drinking rates in the Network Support intervention group reported up to 20% more days abstinent than the other groups (p=0.02), with abstinence rates in the Network Support intervention group reaching 40% at up to 27 months follow-up. Similar to the study by Wingood et al [34] education sessions taught the participant skills to help the patient change their social support network to be more supportive of abstinence and less supportive of drinking, and this was supplemented by attendance at Alcoholics Anonymous (AA).

The intervention developed and tested by Graham et al [38] involved so-called ‘Interrogators’ (i.e. highly engaged ‘seeds’) in an online environment encouraging participants to integrate into a virtual smoking cessation community by engaging with other community members. The intervention attempted to influence the formation of social ties in an online network using some of its existing members. The study did demonstrate significantly greater use of the website and increased social support compared to a web only control group (p<0.01). However, the study did not collect social network data that would enable us to discern whether the intervention actually resulted in the formation of new social ties.

One study involved using information from personal network diagrams to enable participants to reflect on supportive (with whom they were encouraged to strengthen ties with) or unsupportive network members (with whom they were encouraged to distance themselves from). Eaton et al [37] tested an intervention where participants (at risk HIV-negative MSM) mapped their own network diagram of sexual partners and used this information to develop a plan they could carry out to reduce their HIV risk. Participants then reflected on instances in which they potentially exposed themselves to HIV, thus creating a teachable moment. Results of the study showed a positive intervention effect at one and three months follow-up.

***Intervention Length***

For sexual health outcomes, subgroup analyses were significant for intervention length at ≤six months (p<0.05; S8 Fig). The results of all other subgroup analyses, including those for drug risk outcomes, were non-significant. See S8—S13 Fig.

***Age***

For sexual health outcomes, subgroup analyses were significant for mean age of participants at ≤six months (p=0.002; S14 Fig), and at 6—12 months (p=0.02; S15 Fig). The results of all other subgroup analyses, including those for drug risk outcomes, were non-significant. See S14—S19 Fig.

***Gender***

For sexual health outcomes reported at last follow-up (i.e. after 12 months post-baseline), the subgroup analyses investigating percentage of female participants (p<0.001; S22 Fig) found significant effects. The results of all other subgroup analyses were non-significant. See S20—S25 Fig.

The forest plots for the sub-group analyses are presented in S2—S25 Fig. The results can be interpreted using the legend below.

Significant effect in favour of Intervention group

Non-significant

Significant effect in favour of Control group
