# Supplementary material for: Social network interventions for health behaviours and outcomes: A systematic review and meta-analysis
Source: PLoS Med. 2019 Sep 3;16(9):e1002890. doi: 10.1371/journal.pmed.1002890 (PMC6719831; doi:10.1371/journal.pmed.1002890)
Supplement: S4 Text — (DOCX) [file pmed.1002890.s005.docx]

**S4 Text: Sensitivity analyses**

For sexual health outcomes reported at ≤six months, sensitivity analyses showed that there was a significantly higher pooled OR for studies with an attrition rate of less than 20% versus studies with an attrition rate of 20% or greater (p=0.002; S38 Fig). For sexual health outcomes reported at ≤six months, sensitivity analyses also showed that there was a significantly higher pooled OR for studies that used randomized controlled trial (RCT) or cluster randomized controlled trial (cRCT) designs versus studies which used any other design (p=0.002; S44 Fig). For sexual health outcomes reported at >6 months to ≤12 months, sensitivity analyses showed that there was a significantly higher pooled OR for studies that used RCT or cRCT designs versus studies which used any other design (p=0.003; S45 Fig). For drug risk outcomes reported at ≤six months, sensitivity analyses showed that there was a significantly lower pooled OR for studies that used RCT or cRCT designs versus studies which used any other design (p=0.0006; S47 Fig).

For all other analyses, the results of all sensitivity analyses showed that the pooled effect size estimate was robust to the omission of studies classified at high risk of bias (S26—S31 Fig), studies not conducting an intention-to-treat analysis (S32—S37 Fig), studies with high attrition rates (S38—S43 Fig) and studies using non-randomized designs (S44—S49 Fig).
