# Supplementary material for: Social network interventions for health behaviours and outcomes: A systematic review and meta-analysis
Source: PLoS Med. 2019 Sep 3;16(9):e1002890. doi: 10.1371/journal.pmed.1002890 (PMC6719831; doi:10.1371/journal.pmed.1002890)
Supplement: S5 Text — (DOCX) [file pmed.1002890.s006.docx]

**S5 Text: Publication bias**

There was little indication of publication bias from inspection of the funnel plots for the other analyses.

*Studies reporting sexual health outcomes*

For sexual health outcomes reported at ≤six months, both the Egger and PEESE methods showed no evidence of small-study effects (p≥0.69). The intercept, representing the best estimate of intervention effect after adjustment for small-study effects, was non-significant using both the Egger's method (1.13; 95% CI: 0.21, 5.98) and the PEESE approach (1.28; 95% CI: 0.60, 2.75). See S50 Fig.

For sexual health outcomes reported at >six months to <12 months, both the Egger and PEESE methods showed no evidence of small-study effects (p≥0.84). The intercept, representing the best estimate of intervention effect after adjustment for small-study effects, was non-significant using both the Egger's method (1.50; 95% CI: 0.64, 3.53) and the PEESE approach (1.47; 95% CI: 0.94, 2.29). See S51 Fig.

For sexual health outcomes reported at last follow-up, both the Egger and PEESE methods showed no evidence of small-study effects (p≥0.60). The intercept, representing the best estimate of intervention effect after adjustment for small-study effects, was non-significant using both the Egger's method (1.24; 95% CI: 0.33, 4.69) and the PEESE approach (1.41; 95% CI: 0.55, 3.66). See S52 Fig.

*Studies reporting drug risk outcomes*

For drug risk outcomes reported at ≤six months, both the Egger and PEESE methods showed no evidence of small-study effects (p≥0.26). The intercept, representing the best estimate of intervention effect after adjustment for small-study effects, was non-significant using both the Egger's method (0.67; 95% CI: 0.20, 2.32) and the PEESE approach (0.89; 95% CI: 0.44, 1.79). See S53 Fig.

Since there were only two studies included in the meta-analysis for drug risk outcomes reported at >six months to <12 months or less, and at last follow-up, there were too few studies to conduct these analyses.

The results of these tests should be interpreted cautiously due to the high degree of heterogeneity.
