# Supplementary material for: Social network interventions for health behaviours and outcomes: A systematic review and meta-analysis
Source: PLoS Med. 2019 Sep 3;16(9):e1002890. doi: 10.1371/journal.pmed.1002890 (PMC6719831; doi:10.1371/journal.pmed.1002890)
Supplement: S1 Table — (DOCX) [file pmed.1002890.s007.docx]

**S1 Table: Social network functions for Individual network interventions**

| **Ref** | **Social Network Definition** | **Network Intervention Strategy** | **Recruitment Strategies** | **Training of Peer Educators/Leaders (where applicable)** | **Social Network Measures and Relevant Characteristics (where applicable)** |
| --- | --- | --- | --- | --- | --- |
| Kelly et al, 1997 [1] | Friends and associates (gay men) visiting specific gay bars in 8 cities | ***1.Individuals (Centrality):***  Recruitment of peer leaders assessed as popular by trained bartenders; those identified asked to identify other popular people as peer leaders; peer training skills for gay men about HIV risk behavior;  ***2.Induction (Network outreach):***  Discussions with peers about HIV risk behavior; peer leaders committed to talk to 4-10 peers before next training session; wore badges at bars that were consistent with HIV risk promotional material to induce discussions  **Theoretical Framework:** Behavioural modelling | To identify popular opinion leaders, bartenders in all the intervention-city bars were trained to observe the crowds in the bar for 10 days and to record the names of the people they judged to be the most popular with the other men (e.g., those who were most often greeted, greeted others most, and seemed well-liked). Each bartender made independent popularity nominations; nomination lists were cross-matched, those whose names appeared on more than one list were identified as opinion leaders and recruited for training; each opinion leader was asked to nominate 1-2 popular friends to attend training sessions | ***Number of training sessions***: 5  ***Duration of training sessions***: 2 hours each, once a week for 5 weeks  ***Characteristics of the trainer(s):*** Members of the research team  ***Training elements:*** Modelling and roleplay exercises to deliver messages about behaviour change, e.g. importance of carrying condoms, discussion of risk-reduction precautions with partners before sex, avoidance of sex when intoxicated, refusal of unwanted sexual coercions; to communicate benefits of change, to correct listeners’ misconceptions about risk; to identify safer-sex practices as socially acceptable norms | Measured number of conversations in 3 weeks following training. All opinion leaders reported an average of 10 conversations about HIV with peers over 3 weeks  Approx. 8% of the total number of men present in each city’s bars during the baseline survey period were trained as peer leaders |
| Latkin et al, 1996/1998 [2] | Sex partners and PIDs defined as the leaders "'risk network" members | ***1.Individuals (influential):***  Recruitment of peer leaders; PIDs asked to nominate leaders and to recruit them to the study  ***2.Induction (network outreach):***  Community outreach for PIDs; peer leaders encouraged to discuss HIV prevention with their network; set goals to have discussions with 1 network member and broader network of family, friends  **Theoretical Framework:** Social influence and empowerment framework | Peer leaders were chosen by a nomination. Nominators were then asked to list individuals they considered leaders in the PID community and to recruit them into the study | ***Number of training sessions***: 10  ***Duration of training sessions***: 90 mins each  ***Characteristics of the trainer(s):*** not detailed  ***Training elements:*** cognitive behavioural skill-building training and outreach education training programme; concepts of social norms and social influence and leadership skills of goal setting, effective communication, modelling, and conflict resolution. Processes of making public commitments to risk reduction, behavioural modelling, skills rehearsal, and behavioural cues in natural settings. At the last session, leaders were asked to bring at least 1 risk network member whom they had listed on their baseline network inventory | Network inventory [a]: participants asked to list members of their personal support network whom they had known for at least 1 month. After naming the members of their support network, participants were asked to list individuals they had known for at least 1 month and with whom they shared injection drugs or had had sex with in the past 6 months. The leaders also were asked whether any of their support network members used injection drugs.  The peer leaders documented 2165 HIV prevention interactions, of which 84% were with active drug users. |
| Sikkema et al, 2000 [3] | Women resident in participating housing developments | ***1.Individuals (influential):***  Selection of peer leaders; nominated 5 'liked most and trusted for advice'  ***2.Induction (network outreach):***  Events run by peer leaders; opinion leaders formed a Women’s Health Council to undertake AIDS prevention outreach activities targeting other women who lived in their development; opinion leaders recruited other residents to sexual health promotion events; posters and brochures placed in the housing development announced the availability of the workshops  **Theoretical Framework:** Not detailed | Each female resident was asked to name up to 5 women living in the same development who she ‘liked most and trusted for advice’; opinion leaders were those who received the greatest number of peer nominations per development (9-14 opinion leaders per development) | ***Number of training sessions***: 4 workshops  ***Duration of training sessions***: 90 mins each  ***Characteristics of the trainer(s):*** 2 female facilitators trained by the research team  ***Training elements:*** HIV and STD risk education, women’s reproductive health and sexuality issues, male and female condom use, sexual assertiveness and negotiation skills regarding condom use, risk behaviour self-management, and skills training to talk with family and friends about HIV and sexual behaviour; stressed the important role that can be played by women who take on leadership in HIV prevention efforts in their community. | Workshops were attended by an average of 48% (range 40%–57%) of all adult female residents  Approx. 12% of all the adult women who lived in each development were recruited as opinion leaders |
| Amirkhanian et al, 2005 [4] | MSM and their measured ‘social circles’ (socializing groups characterised by stability, close proximity, and frequent positive observations) | ***1. Individuals (influential***):  Selection of peer educators; each index identified up to 9 network members who were then asked to score leadership (psychological support; trust; discussion of important matters) for each member  ***2.Individuals (centrality):***  Recruitment of index participants; observed by research staff to be central to a stable group at bars  ***3.Induction (leaders within groups):***  Discussions with network members; leaders were trained in knowledge and communication skills and encouraged to talk to friends between sessions.  ***4.Induction (RDS):***  Indexes provided the 1^st^ names of up to 9 of their own (egocentric) network members  **Theoretical Framework:** Not detailed | Ethnographers located and systematically observed all bars and nightclubs attended by young MSM in both cities, and selected venues in which ‘social circles’ could be identified. Social circles observed to establish initial contacts with ‘index’ individuals who could serve as access points to high-risk social networks. Each circle’s ‘index’ (person who was its social and affective centre of attention) was identified and provided with study information.  Indexes provided the 1^st^ names of up to 9 of their own (egocentric) network members, defined as the friends with whom they most liked to spend time, talk and felt closest among those who were part of their ‘gay life’. Indexes then invited those people to participate in the study. The network member with a high ‘social status score’ was designated as its leader | ***Number of training sessions***: 9 (5 weekly group sessions with 4 booster sessions over next 3 months)  ***Duration of training sessions***: 3-4 hours each  ***Characteristics of the trainer(s):*** Facilitated by 2 experienced psychologists or sociologists  ***Training elements:*** Peer leader attended a group training program with 5-9 leaders of other networks; provided training and guidance in delivering ongoing theory-based HIV prevention advice to other network members. Peer educators taught to include messages in naturally-occurring conversations and tailor messages to particular risk issues of each friend | Network members’ ‘social status’ score was calculated: Ranked individuals within a network based on their balance of positive and negative nominations received from other members across all leadership areas.  Network mean size=5.3 members; 50% of approached indexes and 93% invited network members participated in the study.  Amount of communication was monitored; leaders talked with each network member at least once weekly on a mean of 3.6 (range 0-7) weeks during the intervention; talk about AIDS with friends nearly doubled (mean 3.5 times at baseline to 6.1 at follow-up) but declined across control group members (4.1 to 3.1 times). |
| Kelly et al, 2006 [5] | Roma men in a Roma settlement with approx. 35 000 residents | ***1.Individuals (influential):***  Network members reported most/least relationships in 5 social status domains;  social networks (3-9 men) recruited together by asking an index (observed to be central) member to identify all members  ***2.Induction (leaders within groups):***  leaders trained in how to advise about reducing HIV risk  **Theoretical Framework:** Not detailed | Field teams (staff who had long carried out Roma community development service programmes) with 2 members conducted systematic ethnographic observations throughout meeting places, including street corners, cafes, disco clubs, and back yards. Field staff looked for the presence of “social circles.” When researchers agreed that a group constituted a social circle, they decided who was the circle’s social and affective centre of attention, termed the “index.” Indexes were the entry points for accessing and recruiting social networks.  Each index was asked to indicate the first names of the friends with whom he most liked to spend time, talk to, and trusted. These people were defined as members of the index’s social network and were contacted and recruited for participation | ***Number of training sessions***: each week for 5 weeks followed by booster sessions every 2 weeks and then every 2 months  ***Duration of training sessions***: 2 hours  ***Characteristics of the trainer(s):*** 2 facilitators  ***Training elements:*** Leaders of each network received training on how to counsel and advise other members of the network on reducing HIV risk behaviour. Leaders attended small group training sessions with 6-7 other leaders | Mean size of social networks including the index was 5.3 men (range 3-9)  Monitoring grids' record conversations with other network members |
| Campbell et al, 2008 [6] | Year 8 students (whole school intervention) | ***1.Individuals (influential):***  Identification of peer leaders; all students in Year 8 asked who they respect, good leaders, look up to  ***2.Induction (network outreach):***  Trained influential students acted as peer supporters during informal interactions outside the classroom to encourage their peers not to smoke  **Theoretical Framework:** Diffusion of innovation theory | To identify influential peer leaders, participants were asked “who do you respect in Year 8 at your school?” “who are good leaders in sports or other group activities in Year 8 at your school?” “who do you look up to in Year 8 at your school?” (asked to name up to 5). | ***Number of training sessions***: 2 day training event  ***Duration of training sessions***: 2 days  ***Characteristics of the trainer(s):*** facilitated by a team of external trainers who were experienced in youth work, led by health-promotion specialists  ***Training elements:*** trained to provide information about short-term risks to young people of smoking and the health, environmental, and economic benefits of remaining smoke-free; develop communication skills, listening skills, expression of feelings and ideas, group work, team building, cooperation and negotiation, ways of giving and receiving information, and conflict resolution; and enhance students’ personal development, including their confidence and self-esteem, empathy and sensitivity to others, assertiveness, decision making and prioritising skills, attitudes to risk-taking, and exploration of personal values; included participatory learning activities such as role plays, student-led research, small group work and discussion, and games | 16% acted as peer supporters; majority of clusters were single sex (81-92%); 48-60% of clusters contained at least one peer supporter; Males 6-78 (mode=8) nominations to be peer supporter; females 6-60 (mode=9); 93% retention of peer supporters  To achieve critical mass of 15% as peer supporters, 17.5% of those with most nominations were invited to recruitment meeting  Conversations logged over 10 week period. |
| Kim et al, 2015 [7] | Members of local village | **1.Individuals (influential):**  Selection of initial participants in each of 32 villages with full network mapped;  groups had 5% initial participants - random, high in-degree, or random nominee of a random person  3 network targeting strategies on the diffusion and uptake of 2 interventions:  ***In-degree:*** targeted the 5% of villagers named as a contact most often by others in their village;  ***Nomination targeted:*** targeted a 5% sample of villagers composed of one randomly chosen friend nominated by each member of a 5% random sample of villagers;  ***Randomly targeted:*** targeted a random 5% sample of villagers  ***2.Induction (network outreach):***  Diffusion of products and information; initial participants given pack with product (multivitamins and/or water purifier tablets) and 4 vouchers, and encouraged to distribute voucher and information to network  **Theoretical Framework:** Network theory; diffusion of innovations theory | Measured the entire social network of each village by asking all residents to identify spouses, siblings, and friends from a photographic census  Name generators were:  1. Who are your brothers and sisters that you are friends with?  2. Who are your best friends that are not your brothers and sisters?  3. Who are you married to, or whom are you living with as a husband or wife?  Each initial (1st-wave) ticket redeemers (1^st^ wave) received a packet of 4 tickets for distribution to other villagers (2^nd^ wave) | ***Number of training sessions***: 1  ***Duration of training sessions***: 1 day  ***Characteristics of the trainer(s):*** Research staff  ***Training elements:*** During the course of 1 day for each village, each targeted individual was delivered an intervention consisting of a health product, instructions for use, and an educational component; targeted individuals were given supplementary information about the interventions and asked to relay this information to others, allowing the diffusion of knowledge to be tracked and product adoption | The same interventions were delivered to the same fraction of the population (5%)  Every ticket was uniquely identified and was signed, dated, and checked by a participating shopkeeper against a list of eligible study participants upon redemption, which enabled tracking of the diffusion of products through the village networks with time |
| Amirkhanian et al, 2015 [8] | MSM and their close friends (3 generations of recruitment waves) | ***1.Individuals (centrality***):  Identification of peer leaders; network members with the highest sociometric scores were first identified. Second, and among them, persons with highest “betweenness centrality” (bridging otherwise unconnected network segments) were selected because they could serve as information exchange channels with others in the network. To ensure intervention exposure, a map of each network was inspected to identify individuals unconnected to the selected leaders, with additional leaders then identified to “cover” these clusters  ***2.Induction (RDS):***  3 generations of nominations of MSM, seeds at venues, their friends, then their friends; included only if at least half of first generation nominees agreed  ***3.Induction (leaders within groups):***  Leaders were trained in giving information and encouraged to talk to friends about HIV prevention; each leader was given the names of network members who had been linked to him based on the sociometric measures; the leader was assigned to talk with these individuals after each session  **Theoretical Framework**: Social Cognitive Theory; Theory of Reasoned Action | To identify seeds, study staff observed “social circles” of MSM interacting with one another in the venues, and the circle's centre of attention was designated as a seed and invited to participate. 18 “seeds” from community venues invited the participation of their MSM friends who, in turn, invited their own MSM friends into the study, a process that continued outward until 18 3-ring sociocentric networks were recruited. Networks were eligible if at least half of 1st-ring members were recruited. | ***Number of training sessions***: 5 weekly group sessions followed by 4 booster sessions spaced over the next 3 months  ***Duration of training sessions***: 3 hours each  ***Characteristics of the trainer(s):*** facilitators were centrally trained and followed a manual.  ***Training elements:*** group sessions attended by 5-11 leaders; guided network leaders in giving personal HIV risk reduction advice to their friends; taught network leaders to communicate messages that incorporated theory-based constructs. Each session focused attention on delivering messages to friends based on a different construct; leaders could tailor advice to the particular risk issues of each friend. The facilitators used behavioral techniques to help network leaders gain skill and comfort in having these conversations. Role plays allowed network leaders to generate examples and rehearse ways to communicate HIV prevention messages to network members. In each session, leaders gave one another feedback to shape their skills, discussed HIV prevention communication experiences in the past week, and were reinforced for their efforts | Participants answered 3 sociometric questions about each friend (i.e. whether they often talk together to discuss important issues)  30% peer leaders participated  Networks ranged in size from 9-65 members (mean=35)  Occurrence of conversations measured from notations that leaders made when completing monitoring grids; participants reported how many times a friend had talked to them about safer sex and about HIV/AIDS in the past 3 months |
| Woudenberg et al, 2018 [9] | School classes | ***1.Individuals (influential):***  Identification of peer leaders; all students in a classroom were asked to complete 7 peer nomination questions on friendship, advice, leadership and peer influence  ***2.Induction (network outreach):***  Trained influential students acted as peer supporters during informal interactions outside the classroom to encourage their peers to be more active  **Theoretical Framework:** Self-determination theory and self-persuasion theory | Influence agents within each classroom were identified with the use of seven peer nomination questions. Three questions were based on the ASSIST-based studies:  friendship, advise, and leadership (Campbell et al, 2008). The remaining four questions were based on peer influence mechanisms involving physical activity (i.e., “with whom do you hang out?”; “To whom do you want to come across as an active person?”; “Who does sports or activities that you also would like to do?”; and “With whom do you talk about physical activity?”). Participants could nominate peers of the same grade, by clicking on their names that were presented in a list on the research smartphone. Participants were free to nominate an unlimited number of peers but were required to nominate at least one other schoolmate. The most central participants were determined based on closeness centrality by entering all the sociometric nominations in the KeyPlayer package | ***Number of training sessions***: N/A  ***Duration of training sessions***: 1  ***Characteristics of the trainer(s):*** Smartphone application  ***Training elements:*** The training adapted elements from a training stimulating healthy drinking behavior used by Smit et al (2016) [b].  The training was based on insights from the Self- Determination Theory and self-persuasion theory. The training consisted of four components: introduction, knowledge, skills and acceptance of the task. The role of the team captain (i.e., influence agent) was explained and questions about their own physical activity were asked to make the topic more salient. Subsequently, the training focused on knowledge about the benefits of physical activity. To raise competence as an influence agent, the influence agents were thought influence strategies to promote physical activity in the classroom. To increase their autonomy, the intervention emphasized that the team captains were free to use one or multiple influence strategies, and were also free to come up with other strategies In the subsequent five days, all team captains received daily reminders on the benefits of physical activity and the four influence strategies | The top 15% of males and top 15% of females in each classroom were identified as influence agents. In total, 24 participants were identified as influence agents. Of the approached influence agents, 19 participants (42% male, age: 12–13 years old) accepted the role, 1 participant declined, and 4 participants did not respond to the invitation. |

References: ^a^ Barrera MA. A method for assessing social support networks in community survey research. Connections 1980;3: 8—13; ^b^ Smit CR, de Leeuw RNH, Bevelander KE, Burk WJ, Buijzen M. A social network-based intervention stimulating peer influence on children’s self-reported water consumption: a randomized control trial. Appetite. 2016;103:294—301.

Abbreviations: AIDS: Acquired Immune Deficiency Syndrome; ASSIST: A Stop Smoking in School Trial; HIV: Human Immunodeficiency Virus; MSM: Men who have sex with men; N/A: Not applicable; PID: person who injects drugs; RDS: Respondent driven sampling; STD: Sexually transmitted disease
