# Supplementary material for: Social network interventions for health behaviours and outcomes: A systematic review and meta-analysis
Source: PLoS Med. 2019 Sep 3;16(9):e1002890. doi: 10.1371/journal.pmed.1002890 (PMC6719831; doi:10.1371/journal.pmed.1002890)
Supplement: S2 Table — (DOCX) [file pmed.1002890.s008.docx]

**S2 Table: Social network functions for Segmentation network interventions**

| **Ref** | **Social Network Definition** | **Network Intervention Strategy** | **Recruitment Strategies** | **Training of Peer Educators/Leaders (where applicable)** | **Social Network Measures and Relevant Characteristics (where applicable)** |
| --- | --- | --- | --- | --- | --- |
| Trotter et al, 1996 [10] | PIDs and their drug networks | **1.Segmentation (cohesive groups):**  1 session delivered to whole (small) network as a group. Emphasised mutual protection and group agree risk management rules. Identified network members were brought together to conduct HIV risk-related problem solving and norm identification for the group, and to provide motivation for the group to take action to protect itself, as well as individual members.  **2.Induction (recruitment and interaction):**  Outreach workers were trained to provide active education and intervention information in the field  **Theoretical Framework**: Not detailed | Chain referral and snowball recruitment strategies: 2 indigenous outreach workers (formerly part of the drug networks) contacted individuals who were bridges into representative drug-use networks. The outreach workers and their contacts identified all network members and attempted to recruit all members into the intervention. Through the course of the project, 50 different networks were recruited, plus a number of isolates or members of networks where only one individual could be recruited | Not applicable | Not detailed |
| Kincaid et al, 2000 [11] | Women who were centrally located in a village and their informal village-based social network | ***1.Segmentation (cohesive group):***  Field health workers delivered education program to small networks; meetings held in the homes of family planning opinion leaders (satisfied current adopters) who were centrally located but geographically dispersed to cover the entire village network, making use of the existing informal social network while at the same time changing the content of communication by providing a forum for the face-to-face discussion of family planning with other women.  **Theoretical Framework**: Social Network Theory; Diffusion of Innovation Theory | Training of field workers to improve their interpersonal communication, counseling, and group leadership skills and to locate and mobilize opinion leaders (`link persons') in each communication network. Identification of village communication networks to locate volunteer link persons who were: centrally located within the social network; satisfied users of family planning and willing to promote it publicly; influential within the social network. Establishment of rotating peer group discussions by each field worker which: met at the homes of local link persons at least once a month; discussed health and family planning issues; encouraged peer group support and influence; included counselling; provided family planning supplies at the meetings | Not applicable | Not detailed |
| Minnis et al, 2014 [12] | Friends who met eligibility criteria (e.g. gang affiliation)**;** Indexes recruited 2 friends | **1.Segmentation (cohesive groups):**  Index and friends randomised together and attended sessions together.  **2.Induction (recruitment):**  Each participant encouraged to recruit 2 friends; discussed session topics with group friends and group members outside the intervention sessions  **Theoretical Framework**: Guided by behavioural economics and social learning theory within a social networks framework | Index participants were recruited through street, community, and school-based outreach strategies. Eligible and interested youth were given 2 referral cards to distribute to friends who might want to enrol in Yo Puedo with them.  Index participants asked to recruit up to 2 friends; 8-session life skills group promoted sexual health with a focus on STI and unintended pregnancy prevention and early childbearing norms. | Not applicable | Composition of participants’ close friends measured. Results found that three quarters of intervention participants who attended 3 or more sessions reported that they became closer with the friends with whom they enrolled. Half made new friends in the groups, and three quarters discussed session topics with group members outside the intervention sessions |
| Shaya et al, 2014 [13] | Friends and relatives with diabetes**;** Indexes invited 3-4 peers (neighbours, friends, family members) | ***1.Segmentation (cohesive group):***  Indexes invited 3-4 peers (neighbours, friends, family members). Education component delivered to egonets of 3 (cluster)  ***2.Segmentation (interaction):***  Education component emphasised team building and information sharing within egonet cluster  **Theoretical Framework**: Social Network Theory | 50 patients enrolled by the study staff (at the medical practices and community sites with University of Maryland) was randomised into either of the 2 groups. 1^st^ wave participants (n=25) recruited by study staff; 2^nd^ wave, invitation extended by Indexes to relatives and friends with diabetes to participate in the study (n=43). Each 1^st^ wave participant, along with the two 2^nd^ wave participants they recruited collaborated as a group, resulting in small social network clusters of 3 participants each.  Indexes formed small clusters of 2-3 friends/relatives and attended monthly diabetes education sessions facilitated by a nurse. Participants were encouraged to engage in team building exercises and share health information with their own cluster, turning it into a supportive group. | Not applicable | Social network connectivity (Social Network Index questionnaire [a] which measures 3 social connectivity metrics—number of high contact roles, number of people in social network and number of embedded social networks).  Number of high contact roles in the social network (0.61, p=0.04 at 3 months; and 0.66, p=0.04 at 6 months), number of people in social network (3.15, p=0.001 at 3 months; and 4.48, p<0.0001 at 6 months), number of embedded social networks (0.62, p=0.001 at 3 months; and 0.65, p=0.001 at 6 months) in the intervention group |
| Cobb et al, 2014 [14] | Adults who were members of an online community; encouraged to invite their ‘real life’ friends and make new ‘friends’ with other members of the online community | ***1.Segmentation (cohesive group):***  Participants encouraged to recruit friends, make friendships and do challenges together  ***2. Alteration (adding nodes):***  Participants encouraged and incentivized to form ‘friend’ connections with other members on the intervention website and form pacts to complete challenges together. Members can form pacts to complete challenges together, encourage one another, cheer each other on via “smiles,” and comment on each other’s challenge completion stories. Engagement was rewarded with points, badges, and other virtual elements drawn from game design work.  **Theoretical Framework**: Social Cognitive Theory | Advertisements were placed within Facebook, running 82 different ads over 47 days. Individuals clicking ads run specifically for the trial (e.g., Do one small action every day to improve well-being. Sign-up is easy and no cost!) were taken to the intervention website. Members were encouraged to recruit individuals from their real-life social network and connect with them within Daily Challenge. | Not applicable | Not detailed |

Reference: ^a^ Cohen S, Doyle WJ, Skoner DP, Rabin BS, Gwaltney JM. Social ties and susceptibility to the common cold. JAMA 1997;277: 1940—1944. Abbreviations: AIDS: PID: person who injects drugs; STI: Sexually transmitted infection
