# Supplementary material for: Social network interventions for health behaviours and outcomes: A systematic review and meta-analysis
Source: PLoS Med. 2019 Sep 3;16(9):e1002890. doi: 10.1371/journal.pmed.1002890 (PMC6719831; doi:10.1371/journal.pmed.1002890)
Supplement: S3 Table — (DOCX) [file pmed.1002890.s009.docx]

**S3 Table: Social network functions for Induction network interventions**

| **Ref** | **Social Network Definition** | **Network intervention strategy** | **Recruitment strategies** | **Training of Peer Educators/Leaders (where applicable)** | **Social Network Measures and Relevant Characteristics (where applicable)** |
| --- | --- | --- | --- | --- | --- |
| Kegeles et al 1996 [15] | Young MSM and their gay friends and partners | ***1.Induction (network outreach):***  Outreach conducted by young MSM who spoke with their peers and encouraged them to engage in safer sex (1) to diffuse the safer sex message throughout their community; (2) to recruit additional men to the project. (a) *Formal outreach* ***-*** young MSM went to locations frequented by young MSM to communicate with and encourage others to about the need for safer sex; *(b) Informal outreach* ***-*** young men communicating with their friends in casual conversations about the need to engage in safer sex. Community social events for young gay men that include safe sex education.  **Theoretical Framework:** Diffusion of innovation theory and social influence | Participants recruited by teams of local MSM who distributed surveys at settings frequented by young MSM including bars and university and community setting and through their informal social network. | ***Number of training sessions***: 1  ***Duration of training sessions***: 3 hours  ***Characteristics of the trainer(s):*** peer led  ***Training elements:*** Peer-led one-time small group meetings (8-10 people) to motivate and train participants to conduct informal outreach with their friends on the need for consistently engaging in safer sex. Participants asked to commit to inviting several friends to a small group discussion. | 15-20% taught informal outreach strategies.  Exposure to intervention activities (number of activities engaged in during the past year, e.g. received outreach, attended a social event). 12% participated in formal outreach; 19% attended small group meetings |
| Latkin et al 1996a [16] | Individuals with whom they shared drugs | ***1.Induction (network outreach):***  Network planning of safer practices and the monitoring and reinforcement of safer behaviours of each member; taught about social influences and social norms.  **Theoretical Framework:** Harm reduction | Indexes asked to bring into the clinic the intervention members of their drug network who they had listed in their network interview. Indexes met with group facilitators to discuss approaches for the indexes could use to talk to their drug networks to encourage their participation. There was no limit to the number of network members an index could bring to the sessions but a minimum of 4 was required. Indexes received $25 for bringing network members. | ***Number of training sessions***: unknown  ***Duration of training sessions***: unknown  ***Characteristics of the trainer(s):*** Former heroin users who maintained contact with and were respected by active drug users  ***Training elements:*** Information regarding recognition of the general risk of HIV/AIDS to PIDs and personal risk of infection; taught assertiveness skills | At baseline, participants completed a personal network interview in which participants were asked to provide the names of individuals with whom they shared drugs (i.e. their drug networks)  Mean of 4 network members per index. A mean and median number of 3 network members, not including the indexes, attended each session. |
| Buller et al, 1999 [17] | Workplace colleagues (informal network cliques derived from social network measures) | ***1. Induction (network outreach):*** Education by peer leaders; peer educators expected to spend approx. 2 hours per week encouraging healthy eating to ‘clique’ members and log those interactions  ***2.Segmentation (cohesive group):*** Selection of peer leaders within each 'clique'; Intervention delivered to pre-defined cliques using network data at baseline. Cliques defined as informal referent group in which co-workers were no more than 2 social-tie lengths away from one another.  ***3.Individuals (other centrality):*** Selection of peer leaders within each 'clique'; peer educators selected on basis of centrality in communication flow within cliques. Social support was given and received, group norms develop, opinion-leadership functions  **Theoretical Framework:** Diffusion of innovation theory | Recruitment occurred by formal work group to obtain collections of employees who had regular informal contact on a weekly basis – identified by senior managers  An employee in each intervention clique was recruited to be a peer educator. These employees were highly central within the clique in terms of communication ties and flow. As such, they were skilled at communication, had strong relationships with their co-workers, were capable of accessing all co-workers, and could be opinion leaders in the group | ***Number of training sessions***: 16 hours over 8 weeks  ***Duration of training sessions***: 16 hours total  ***Characteristics of the trainer(s):*** Highly central employees in each clique  ***Training elements:*** Included presentations,  group discussions, and role playing to covering: (a) health benefits of eating fruits and vegetables; (b) cultural trends in dietary practices; (c) methods of incorporating the topic of fruits and vegetables in informal communication at work, gaining compliance, and motivating behavior change; and (d) their role and responsibilities. Peer educators were told that the project expected them to spend approx.2 hours/week discussing eating fruits and vegetables with co-workers. Peer educators were taught 5 persuasive communication strategies and ways to initiate informal conversations about fruits and vegetables | Network data were collected via a sociometric question in which respondents were asked to name up to 8 co-workers in their work group with whom they talked. Respondents were then asked to rate each named person on five social-tie dimensions: 1) contact frequency; 2) personal relationship; 3) frequency of health- and diet-related conversations; 4) respect for co-worker’s opinions about health-related information; 5) frequency of eating lunch together  Median clique size at baseline=11 (range 5-18) |
| Wing and Jeffrey, 1999 [18] | Overweight participants and up to 3 friends (friends, co-workers or family members) who wanted to lose weight and were 15-70lb (6.8-31.8kg) overall ideal body weight | ***1.Induction (network outreach):***  Sessions delivered to groups with all 4 network members in same group; meetings led by a behaviour therapist, a nutritionist or both; half the networks also had group building activities within their intervention  ***2.Induction (RDS):***  Participants invited up to 3 of their friends, family, co-workers to form a team and participate in the study  **Theoretical Framework:** Not detailed | Newspaper adverts; asked potential participants whether they and their friends (friends, co-workers or family members) would like to lose weight. All participants and network members had to be 15-70lb (6.8-31.8kg) overall ideal body weight | ***Number of group sessions***: 16 (one per week)  ***Duration of group sessions***: unknown  ***Characteristics of the group facilitator(s):*** Behaviour therapist or nutritionist or both  ***Programme elements:*** Individual weight-in; review of self-monitoring records; lecture or discussion period. Topics included problem solving, assertion, stimulus control, developing social support, dealing with high-risk situations, cognitions, strategies for long-term maintenance | At follow-up, participants were asked to indicate how supportive other study participants had been of their weight loss efforts range from 1(not at all supportive) – 5 (very supportive); frequency with which they spoke to other group members, exercised with them, ate out with them, or shared info related to weight control outside of the group (1=never; 2=occasionally; 3=frequently), using the Sallis Social Support Scale for Eating and Exercise Behaviour questionnaire^1^ |
| Elford et al, 2001 [19] | Gay men who attended the same gym | ***1.Induction (network outreach):***  Leaders identified by gym managers and trained; after training, peer educators were asked to talk to at least 20 gay men in their gym over the next 5 months about HIV risk reduction  ***2. Individuals (influential):***  Peer leaders/educators identified by gym managers  **Theoretical Framework:** Diffusion of Innovation Theory | Gym managers were asked to identify potential peer educators. Of the 144 popular opinion leaders identified by gym staff, 46 (32%) underwent training but only 27 (19%) remained with the project throughout the intervention period | ***Number of training sessions***: unknown  ***Duration of training sessions***: unknown  ***Characteristics of the trainer(s):*** unknown  ***Training elements:*** unknown | Information was collected on the number of men who spoke to a peer educator in their gym. (80%) thought it was useful to have peer educators in the gym to talk about risk reduction and half the men were aware of their presence.  Only 3% said they had spoken to a peer educator during the intervention period. Consequently, the critical mass required for diffusion was not established change in the proportion of men reporting an outcome in the intervention |
| Earp et al, 2002 [20] | Trained lay health advisors and women they knew in their community | ***1.Induction (network outreach):***  Lay health advisors to encourage mammography; trained advisors had approx. 2 conversations per week with network members (women they knew). Community activities (approximately 2 per month) included presentations made to local community groups (at beauty parlors, nutrition sites, churches, and other places where women gathered) and community events (such as health fairs, parades, and mobile mammography van days)  **Theoretical Framework:** Social–ecological model of behavior | 4 community outreach specialists (indigenous community leaders hired and paid by the project but located in local health departments and rural health centres) linked lay health advisors to the health care system. Community outreach specialists and staff recruited and trained 149 lay health advisors in the 5 intervention counties over a period of 18 months. An additional 21 lay health advisors were trained | ***Number of training sessions***: 3-5 sessions  ***Duration of training sessions***: 10-12 hours total  ***Characteristics of the trainer(s):*** Community outreach specialists  ***Training elements:*** Didactic methods, role playing, and other techniques; instruction about breast cancer, breast cancer screening | Not detailed |
| Flowers et al, 2002 [21] | Men in gay bars and their peers | ***1.Induction (network outreach):***  Explicitly based on diffusion, with the intent of promoting secondary discussion in the broader gay men community; discussions with gay men at bars about sexual health. NB: Did not replicate the ‘opinion leaders’ model of Kelly as did not get sufficient numbers so instead paid and recruited people who were peers but not necessarily the ‘most popular’  **Theoretical Framework:** Diffusion of Innovation Theory | 5 exclusively gay bars in each city (commercial gay scene). Bars were surveyed over a 4-week period in each city. All men present or entering the bar were approached to complete a baseline questionnaire and given information about the study | ***Number of training sessions***: 2 days  ***Duration of training sessions***: 2 days  ***Characteristics of the trainer(s):*** unknown  ***Training elements:*** communication skills, role playing approaching men and specific message delivery; they distributed sexual health promotion leaflets in bars; engaged in focused interaction with men on a variety of sexual health issues | 42 peer educators (38 men+4 woman); recruited via commercial gay venues and existing voluntary HIV-related organisations  Direct contact with intervention: At follow up, 59% of intervention group reported Hep B vaccination compared with 44% at baseline.  Peer educators recorded a total of 1484 interactions of, on average, 10 mins duration throughout the intervention period |
| Latkin et al, 2003 [22] | PIDs or weekly contact with PIDs and their sex and drug partners, family and friends, and other community members | ***1.Induction (network outreach):***  Peers educators were encouraged to conduct HIV education and advocate risk reduction among their sex and drug partners, family and friends, and other community members; emphasising social responsibility.  **Theoretical Framework:** Social Identity Theory, Social Cognitive Theory; Social Influence | Recruitment areas in Baltimore City were identified through ethnographic observations, focus groups, and geographical coding of drug-related arrests in Baltimore in the prior 3 years. Frequency of interactions with drug users and willingness to conduct outreach HIV education among drug users was asked. Eligibility criteria included that the participant be ≥ 18 years old; have at least weekly contact with drug users, be willing to conduct AIDS outreach education; be willing to bring into the clinic 2 network members for assessment | ***Number of training sessions***: 10  ***Duration of training sessions***: 90 mins each  ***Characteristics of the trainer(s):*** Male and female indigenous para-professional facilitators  ***Training elements:*** small-group sessions incorporated cognitive–behavioral intervention components focusing on sexual risk reduction and drug risk reduction among risk networks. Training techniques included modeling, practicing, giving and receiving feedback, and public goal setting. Participants asked to make public commitments to increase their own health behaviors and to promote HIV prevention among their networks and community contacts. | Participants were asked at 6 month follow-up whether they had talked to the following types of individuals about HIV in the prior month: (a) family members, (b) sex partners, (c) non-drug users, (d) drug users, and (e) “drug buddies.” *Drug buddies*, or “running buddies,” is a colloquial term referring to individuals who work together to acquire drugs or money. On the network inventory, after delineating the names of social support and drug and sex network members, participants were asked to indicate which network members, if any, they (a) talked to about high-risk drug behaviour, (b) talked to about using condoms, (c) provided with condoms, and (d) provided with bleach to disinfect their syringes. At the 6-months, participants in the intervention group were sig more likely to report talking about HIV in the prior month with family members (35% vs. 19%); sex partners (34% vs. 18%); non-drug users (35% vs. 22%); and drug users (39% vs. 23%) (P<0.05) |
| Morisky et al, 2004 [23] | Work colleagues | ***1. Induction (network outreach):***  Peer educators encouraged to discuss sexual health with their work colleagues. Following a training session, peer counsellors were expected: to educate at least 10 of their peers on STI/HIV/AIDS prevention; to meet with the site coordinator regularly to report the progress of their work; to conceptualize and develop educational materials on STI/HIV/AIDS, including posters, stickers and photonovellas; and to distribute materials on STI/HIV/AIDS to their co-workers  **Theoretical Framework:** Empowerment Theory | All males in each targeted site was invited to participate; 10–20 peer counsellors from each target group were recruited either by unanimous choice of the group, volunteering, or recommendation by their supervisors | ***Number of training sessions***: 1 day  ***Duration of training sessions***: 1 day  ***Characteristics of the trainer(s):*** unknown  ***Training elements:*** review of the technical aspects of STI/HIV/AIDS transmission and control; discussion of some of the myths about STI/HIV/AIDS and clarification of issues; skill-building teaching methods and strategies; counselling techniques; how to prepare Information, Education and Communication materials | Not detailed |
| Garfein et al, 2007 [24] | Young PIDs and their peers in their community | ***1.Induction (network outreach):***  Teaching participants how to educate peers about HIV and HCV risk reduction  **Theoretical Framework**: Social Learning Theory; Information, Motivation, and Behavioral Skills Model | Recruited through street outreach, advertising, and coupon-based participant referrals | ***Number of training sessions***: 6 over 3 weeks  ***Duration of training sessions***: 2 hours each  ***Characteristics of the trainer(s):*** 2 facilitators  ***Training elements:*** Education re: HIV and HCV transmission, described the vital role peer educators play in preventing further disease spread; peer education about safer injection and sexual practices; negotiation skills; role-playing; large group debriefing, goal-setting to encourage continued risk reduction | Not detailed |
| Valente et al, 2007 [25] | Peers in same school class | ***1.Individuals ((influential):***  Identified peer leaders for group activities; participants allocated to group with their closest nominated leader. Networks also mapped for calculation of social norms.  ***2.Induction (network outreach/leaders within group):***  Expected ongoing effect of intervention;  While expected to have ongoing influence, there is no role for the leaders outside the group activities.  **Theoretical Framework**: Social Influence | At baseline, students were asked to indicate 5 students who make the best leaders for a project in class. Peer leaders were identified as those students who received the most nominations | ***Number of training sessions***: unknown  ***Duration of training sessions***: unknown  ***Characteristics of the trainer(s):*** unknown  ***Training elements:*** taught how to facilitate group discussion, how to manage group interaction and encouraged to embrace anti-substance use norms. Normative restructuring about drug use was an essential part of the peer leader training | At baseline, students were asked to indicate 5 students who make the best leaders for a project in class (using a roster, name 5 classmates you consider a friend). Peer leaders were identified as those students who received the most nominations.  Students were asked to indicate 5 students who they would like to be grouped for group projects in class. Groups were created by assigning students to leaders they chose. If none of the leaders they chose were selected as leaders they were assigned to the leader to whom they were sociometrically closest. On average, students provided the names or initials of 4.16 (SD 1.32) friends, and about 1.82 (SD 1.82) of these were friends from the same school. |
| Latkin et al, 2009 [26] | PIDs and their drug and sex networks | ***1.Induction (network outreach):***  Peer educators, who were trained to act as role models in their networks, would increase the salience of risk reduction norms and diffuse proscriptive and descriptive risk reduction norms and behaviours.  ***2.Induction (RDS)***  Index participants were required to recruit at least one risk network member into the study.  **Theoretical Framework**: Bounded Normative Influence; Social influence; Diffusion of innovations; Social Learning; Social Identity; Social Norms | Recruiters arranged community meetings to explain the project. They also provided educational and recreational activities to build a relationship with the community. Throughout the recruitment process, the recruiters held focus groups with PIDs to evaluate recruitment approaches. Index participants were required to recruit at least one risk network member into the study. | ***Number of training sessions***: 6 over 4 weeks and 2 booster sessions at 6 and 12 months  ***Duration of training sessions***: 2 hours each  ***Characteristics of the trainer(s):*** unknown  ***Training elements:*** small group, network oriented peer educator training sessions. Sessions included instruction in methods of harm reduction, developing and practicing communication skills and strategies, roleplays, and problem solving exercises. At each session, participants developed a plan about how they would discuss and encourage injection and sexual risk reduction with the specific network members that they had identified in the network inventory.  Motivational exercises were included to foster and sustain the indexes’ interest in conducting peer education. They discussed their HIV prevention conversations and the communication techniques they used. | A social network inventory was used to identify risk network members with whom the index had regular interactions, for recruitment by the index. Participants were asked to list network members whom they had known for at least 1 month. Asked to list support network members; ‘‘Who are the people that you do drugs with?’’ ‘‘Have you had sex in the last 6 months (even if it wasn’t with your primary partner)? Of the people that you listed so far [on the network] with whom did you have sex within the last six months?  Index members in the intervention arm engaged in more conversations about HIV risk following the intervention compared to control indexes.  The average size of networks enrolled was 3.00 (SE 0.08) in US and 2.35 (SE 0.05) in Thailand. For index members, the median network size identified on the social network inventory was 6 (range 2-21); in US, the median drug network size was 4 (range 0-20). In Thailand, median index network size was 7 (range 3-20), and median drug network size was 4 (range 1-17) |
| Sutcliffe et al, 2009 [27] | Index and drug and/or sex network members in the past 3 months | ***1.Induction (network outreach):***  Indexes trained in methamphetamine-related risk reduction behaviors that they then communicated to members of their social network. No details as to how Index participants were chosen/recruited.  ***2.Induction (RDS)***  Indexes invited network members to participate in the study, identified via a network inventory at baseline  **Theoretical Framework:** Diffusion of Innovation Theory; Social Learning Theory; Social Identity Theory | Not detailed | ***Number of training sessions***: 7 sessions over 1 month and 2 booster sessions at 3 and 6 months  ***Duration of training sessions***: 2 hours each  ***Characteristics of the trainer(s):*** 2 facilitators per session who were in their early  20s and had been a part of the ethnography team in the study's 1st phase.  ***Training elements:*** Taught communication skills to convey methamphetamine and risk reduction messages to social network members. Build group cohesion and identity, social influences in initiating methamphetamine use, sexual risk reduction, STIs, and communication skills in sexual situations; stigma and examined methamphetamine's effects on participants' families and the broader community; community project; peer education homework in which participants would discuss a specific issue with specific peers | Network survey included questions about the number and types of members involved in each participant's social, sexual, and drug networks and their level of interaction and connectedness |
| Tobin et al, 2011 [28] | PIDs and high risk network members. Indexes were asked to invite up to 5 high risk network members (drug users and/or sex partners) | ***1.Induction: network outreach (cohesive group)***  Promoting risk reduction within personal risk networks; 5 group-based, one individual and one session with the Index participant and their enrolled network member (dyad session); sessions focused on increasing knowledge and skills to reduce injection, drug splitting, and sex risk and communication skills to conduct outreach to personal risk networks. The dyad session included an opportunity for the Index to teach the network member about HIV risk reduction options and goal setting for risk reduction. Up to 5 network members invited, but mean was approx. 1.5.  ***2.Induction (network outreach):***  Informal discussion with network members about HIV related topics, hepatitis and responding to overdoes, showing needless syringes to drug buddies  **Theoretical Framework:** Social Identity Theory | Index recruitment: street-based outreach, word of mouth, advertisements and referrals from community agencies. During the Index’s baseline interview, a list of high risk network members who were potentially eligible was generated from a social network inventory. Participants identified people in their personal network based on various roles and functions (e.g. provide social support, sex partner, drug partner). Participants then described characteristics about each individual listed, e.g. gender, age, race, emotional closeness, and frequency of contact. | Not applicable | Index HIV outreach behaviours (examined 4 topics of communication with drug buddies within the prior month: 1) cleaning needles with bleach, 2) risks of sharing needles, 3) using condoms and 4) getting tested for HIV. The number of topics was summed for a total possible score of 4).  Indexes in the intervention group were sig more likely to talk about HIV related topics in the past month and show a needless syringe to drug buddies |
| Bastian et al, 2013 ^[29]^ | Patients with lung cancer and current smokers who were family members or close friends | ***1.Induction (RDS)***  Recruiting network members of lung cancer patients to smoking quit intervention  **Theoretical Framework:** Transactional model of stress and coping | Patients who had at least one family member or close friend who smoked were asked to complete a patient survey to enumerate smokers in their social network (i.e., defined as relatives and close friends of the index patient). Any family members or close friends who smoke including immediate, birth and extended family, as well as anyone the patient perceived as family, such as close friends. Categorized as: immediate family (spouse/partner, daughter and son); birth family (parent, sister, and brother), extended family (niece, nephew, cousin, grandchild, aunt, uncle or grandparent, in-laws (sister or brother, son or daughter), spouse of extended family and spouse's family); and close friend (reported to be “like family” by the patient). | Not applicable | 38% were immediate family members.  Frequency of contact with the patient: whether or not they talked to the patient on the phone weekly or more vs less frequently;  Quality of relationship: using 3-item closeness and 3-item conflict scale (1-4 likert scale) |
| Hoffman et al, 2013 [30] | PIDs and their drug and/or sexual networks | ***1.Induction (network outreach):***  Indexes discussed and modelled injection and sexual risk reduction behaviors with their risk network members. Only index participants received the intervention and the network members were educated by the indexes.  **Theoretical Framework:** Social Influence; Diffusion of Innovations Theory; Social Learning Theory; Social Identity Theory; Cognitive Dissonance; Social Norms | Indexes recruited from a variety of sources e.g. needle exchange sites. Each index brought a mean of 1.3 network participants into the study with a range of 1–4. | ***Number of training sessions***: 8 training sessions (7 group sessions and 1 individual training)  ***Duration of training sessions***: unknown  ***Characteristics of the trainer(s):*** unknown  ***Training elements:*** develop communication skills and encourage participants to share HIV prevention information with their social network members, primarily with sexual and injection partners; invited to repeat (booster) meetings once a month for 4 months to discuss their strategies, successes and failures in implementing information transfer to their network members | Not detailed |
| Gostsis et al, 2013 [31] | Friends or family of the ego | ***1.Induction (WOM)***  Participants recruited alters to assist them with motivation.  **Theoretical Framework:** not detailed | Eligible participants became egos in the study and were instructed to fill out the online referral form, nominating members of their social network to join their ego-network group and enroll in the study as their alters | Not applicable | Egos recruited an average of 1.63 alters to be their “wellness partners”. The minimum and modal group size was 2 (63%) with 13 groups including 3 people (24.1%) and maximum group size was 8 |
| Booth et al, 2016 [32] | PIDs and 2 of their network members (injection network) | ***1.Induction (network outreach)***  Index participants (peer leaders) trained to become educators within their injection network  ***2.Induction (RDS)***  Index participants invited 2 network members to participate in the study  **Theoretical Framework:** Theories of social norms, cognitive dissonance, social diffusion, social identity, and role theory | Outreach workers (recovering drug users) employed by NGOs recruited “index” participants; indexes were required to bring in 2 members of their injecting network to be eligible | ***Number of training sessions***: 5 over 2 weeks  ***Duration of training sessions***: 90 mins each  ***Characteristics of the trainer(s):*** unknown  ***Training elements:*** small groups; skills training in how to teach HIV risk reduction behaviours to network members effectively; encouraged to model safe behaviours with their network members. At the end of each session (lasting about 90 min), peer leaders were provided outreach assignments to do with their network members. In the subsequent session they were asked to discuss their experiences, with the group helping to address issues that might have arisen. | Not detailed |
| Cobb et al, 2016 [33] | Registered users of Facebook and their Facebook friends | ***1.Induction (RDS)***  Uses the network to recruit participants (induction)  ***2.Induction (interaction)***  Uses the network to motivate participants (interaction). Manipulated Facebook smoking cessation app intensity and features to assess effect on diffusion of, and interaction with, app  **Theoretical Framework:** Social contagion | Recruited initial adopters (“study seeds”) via Facebook advertising and earned media | Not applicable | Egolevel social network metrics hypothesized to affect diffusion, including degree (the number of friends as reported by Facebook), the average local clustering coefficient (how tightly knit the personal network), and betweenness centrality (the relative importance of individuals within their own network, normalized for degree) |

Reference: Sallis JF, Grossman RM, Pinski RB, Patterson TL, Nader PR. The development of scales to measure social support for diet and exercise behaviors. **Prev Med 1987;16:**825—836.

Abbreviation: AIDS: Acquired Immune Deficiency Syndrome; Hep B: Hepatitis B; HCV: Hepatitis C virus; HIV: Human Immunodeficiency Virus; MSM: Men who have sex with men; NGOs: Non-governmental organisation; NUID: Non-injection drug users; PID: person who injects drugs; RDS: Respondent driven sampling; SD: Standard deviation; SE: Standard errors; STI: Sexually transmitted infection: US: United States; WOM: Word-of-mouth
