# Supplementary material for: Social network interventions for health behaviours and outcomes: A systematic review and meta-analysis
Source: PLoS Med. 2019 Sep 3;16(9):e1002890. doi: 10.1371/journal.pmed.1002890 (PMC6719831; doi:10.1371/journal.pmed.1002890)
Supplement: S4 Table — (DOCX) [file pmed.1002890.s010.docx]

**S4 Table: Social network functions for Alteration network interventions**

| **Ref** | **Social Network Definition** | **Network Intervention Strategy** | **Recruitment Strategies** | **Training of Peer Educators/Leaders (where applicable)** | **Social Network Measures and Relevant Characteristics (where applicable)** |
| --- | --- | --- | --- | --- | --- |
| Wingood et al, 2004 [34] | Women with HIV and their network members (biologically related kin or non-kin who provided practical, informational or emotional social support) | ***1.Alteration (adding and deleting nodes):***  Education/training sessions included assisting women to identify people in their social network who had provided social support and in recognising the essential qualities of supportive network members.  **Theoretical Framework:** Social Cognitive Theory; Theory of Gender and Power | Project staff recruited participants from 7 clinics and health departments in Alabama and Georgia. After receiving care from their HIV/AIDS clinic providers, women were referred to project recruiters who screened them for eligibility (female; 18-50yrs; sexually active in past 6 months) | ***Number of training sessions***: 4 sessions  ***Duration of training sessions***: 4 hours  ***Characteristics of the trainer(s):*** Facilitated by female trained health educator and HIV-positive female peer educator  ***Training elements:*** Discussed ways of maintaining supportive network members, encouraged women to seek new network members, and informed participants how to disengage from unsupportive network members. Also taught communication skills for negotiating safer sex, distinguishing healthy and unhealthy relationships. | Network structural factors assessed: Number of network members providing practical support (loaning money, taking care of participant; providing help in an emergency); informational support (offering info and advice about medications/health issues); emotional support (expressed empathy, share their feelings with, supportive re: problems). These were summed to give a total number of network members providing social support. |
| Litt et al, 2007 [35]; 2009 [36] | Social networks of drinkers particularly friends, families and acquaintances **(**people with whom they spent the most time with in the last 12 months) | ***1.Alteration (adding nodes):***  Emphasis on manipulating social network to discourage alcohol use by identifying, expanding and mobilizing the social networks of drinkers. Number of drinking friends same but new non-drinking friends added. Sessions to help the patient change their social support network to be more supportive of sobriety and less supportive of drinking; attendance at Alcoholics Anonymous (AA)  **Theoretical Framework:** Social Network Theory | Participants were recruited through newspaper/radio advertisements. Eligibility: ≥ 18 years old, meet  Diagnostic and Statistical Manual of Mental Disorders (DSM–IV) criteria for alcohol dependence or abuse. | ***Number of training sessions***: 12 sessions  ***Duration of training sessions***: 1 hour  ***Characteristics of the trainer(s):*** unknown  ***Training elements:*** unknown | Important People and Activities instrument (IPA)^1^ to measure network support for drinking and for abstinence. Asks patients to identify important people in their social network, defined as those people with whom they spent the most time in the previous 12 months. For each person identified, the patient specifies the nature of the relationship (e.g., spouse, brother, friend, co-worker), the duration of the relationship, the frequency of contact, the drinking behaviour of each person (frequency and quantity), and the person’s behaviour with respect to the patient’s drinking (supportive, neutral, non-supportive of drinking, or supportive of abstinence). |
| Eaton et al, 2011 [37] | Sexual partners during the last 6 months | ***1.Alteration (education re: social network rewiring)***  Participants were asked to create their own sexual network diagram by providing information about their sexual partners and acts during the last 6 months. Participant diagrams were compared with the character’s diagram, thereby allowing the participants to observe how their behaviours related to those of an evidence-based character who tests HIV positive. Through this activity, participants readily reflected on instances in which they potentially exposed themselves to  HIV, thus creating a teachable moment.  Participants used their own sexual network diagram as a guide to forming a plan they could carry out to reduce their HIV risk  **Theoretical Framework:** Conflict Theory of Decision-making | Flyers were placed at HIV testing sites, treatment centres, and gay identified venues such as bars, bathhouses, and clubs. Advertisements were placed in local gay newspapers and on an Internet classifieds website | Not applicable | Not detailed |
| Graham et al, 2016 [38] | Members of online community trying to quit smoking | ***1.Alteration (add/strengthen desirable edge)***  New participants welcomed into online smoking cessation community and encouraged to engage with other community members trying to quit smoking by ‘Interrogators’. The intervention attempted to influence the formation of social ties in an online network using some of its existing members    **Theoretical Framework:** Implementation Science Framework | Participants were new registered users of BecomeAnEX.org, a free, publicly available smoking cessation website. | “Integrators” served as a “welcome wagon” to expose new study participants to the nature of the community and to encourage them to browse. Participants received proactive communications from 3 established members of BecomeAnEX (“Integrators”) who were longstanding, very active members of the community. Within 24 hours of study enrollment, the Integrators posted a public message on a new member’s personal BecomeAnEX profile page (“wall”) to welcome them, encourage them to fill out their profile, or comment on some aspect of their profile. Integrators did not receive any formal training in cessation treatment and were instructed not to address questions or comments specifically about cessation other than to encourage participants’ efforts and direct them to relevant content and tools on the Web site. | Not detailed |

Reference: Longabaugh R (2001). Manual for the administration of the Important People Instrument adapted for use for BST Decision Trees, Center for Alcohol and Addiction Studies, Brown University, Providence, RI.

Abbreviations: AA: Alcoholics Anonymous; AIDS: Acquired Immunodeficiency Syndrome; HIV: Human Immunodeficiency Virus; IPA: Important People and Activities instrument
