# Supplementary figures and images for: Social network interventions for health behaviours and outcomes: A systematic review and meta-analysis
Source: PLoS Med. 2019 Sep 3;16(9):e1002890. doi: 10.1371/journal.pmed.1002890 (PMC6719831; doi:10.1371/journal.pmed.1002890)

**S1 Fig: Risk of bias of included studies**

**
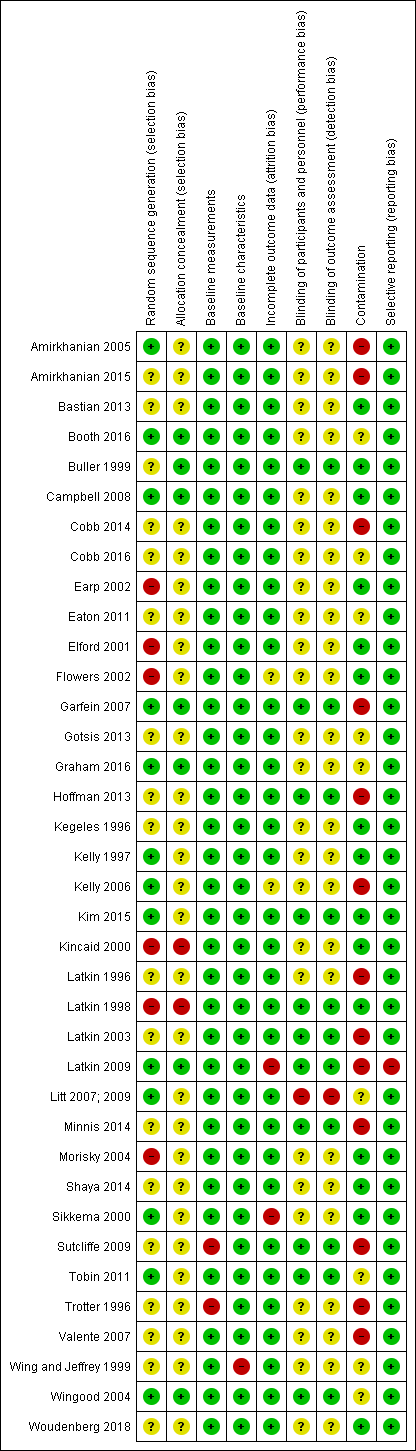
**

**
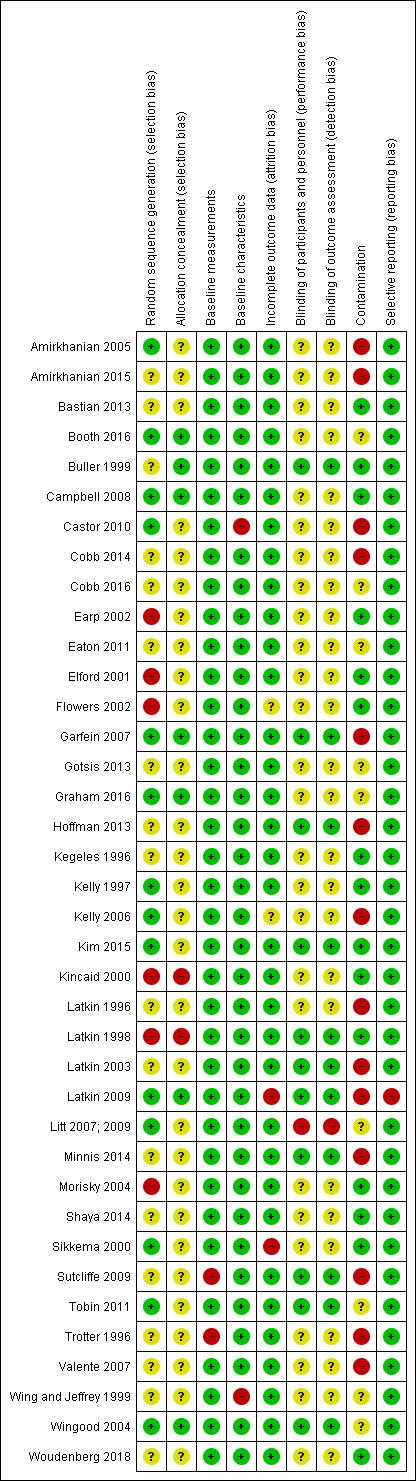
**


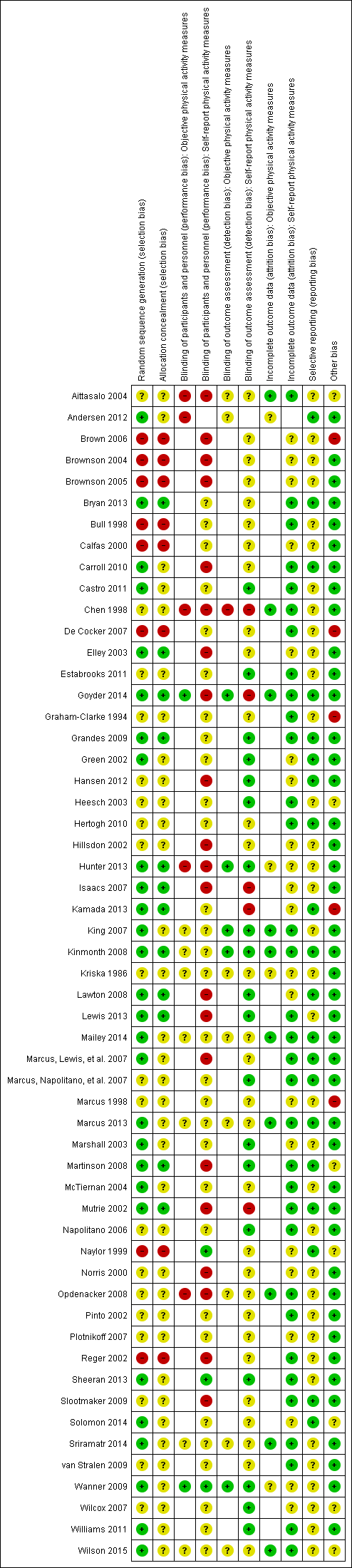
 Low Risk of bias


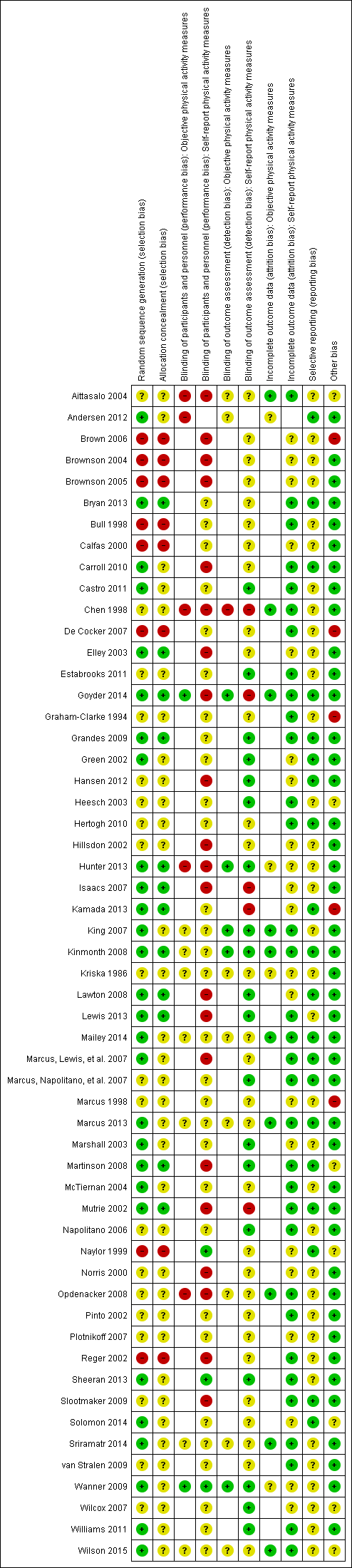
Unclear risk of bias


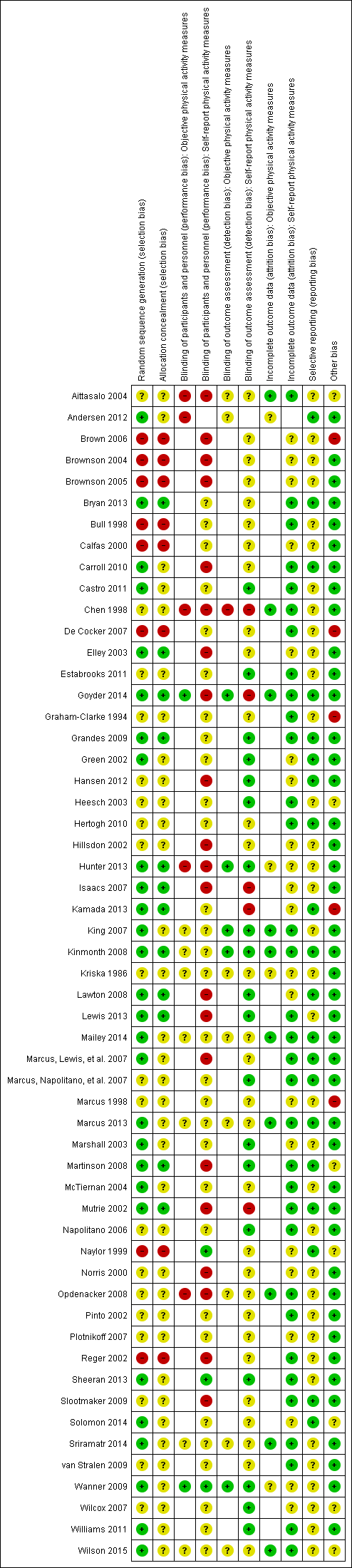
High risk of bias

Supplement: S1 Fig — (DOCX) [file pmed.1002890.s011.docx]
