# Supplementary material for: Social network interventions for health behaviours and outcomes: A systematic review and meta-analysis
Source: PLoS Med. 2019 Sep 3;16(9):e1002890. doi: 10.1371/journal.pmed.1002890 (PMC6719831; doi:10.1371/journal.pmed.1002890)
Supplement: S2 Fig — (DOCX) [file pmed.1002890.s012.docx]

**S2 Fig: Forest plot for subgroup analysis of sexual health outcomes reported at** ≤**six months: intervention approach (individual, segmentation, induction, alteration)**

Favours Intervention

Favours Control

| **Intervention approach** |  | **Odds ratio (95% CI)** | **I-squared (%)** |
| --- | --- | --- | --- |
| Individual |  | 2.09 (1.63, 2.67) | 0 |
| Segmentation |  | 2.38 (0.80, 7.11) | NA |
| Induction |  | 0.76 (0.52, 1.11) | NA |
| Alteration |  | 1.09 (0.79, 1.51) | 0 |
|  |  |  |  |
|  |  |  |  |
|  |  |  |  |
