# Supplementary material for: Social network interventions for health behaviours and outcomes: A systematic review and meta-analysis
Source: PLoS Med. 2019 Sep 3;16(9):e1002890. doi: 10.1371/journal.pmed.1002890 (PMC6719831; doi:10.1371/journal.pmed.1002890)
Supplement: S3 Fig — (DOCX) [file pmed.1002890.s013.docx]

**S3 Fig: Forest plot for subgroup analysis of sexual health outcomes reported at >six months to <12 months: intervention approach (individual, segmentation, induction, alteration)**

| **Intervention approach** |  | **Odds ratio (95% CI)** | **I-squared (%)** |
| --- | --- | --- | --- |
| Individual |  | 1.62 (1.35, 1.95) | 0 |
| Segmentation |  |  | NA |
| Induction |  | 1.36 (0.80, 2.32) | 80 |
| Alteration |  | 1.29 (0.86, 1.91) | NA |
|  |  |  |  |
|  |  |  |  |
|  | Favours Intervention  Favours Control |  |  |
