# Supplementary material for: Social network interventions for health behaviours and outcomes: A systematic review and meta-analysis
Source: PLoS Med. 2019 Sep 3;16(9):e1002890. doi: 10.1371/journal.pmed.1002890 (PMC6719831; doi:10.1371/journal.pmed.1002890)
Supplement: S8 Fig — (DOCX) [file pmed.1002890.s018.docx]

**S8 Fig: Forest plot for subgroup analysis of sexual health outcomes reported at** ≤**six months: intervention length (3 months or less; 3 months—6 months or less; 6 months or longer)**

Favours Intervention

Favours Control

| **Intervention length** |  | **Odds ratio (95% CI)** | **I-squared (%)** |
| --- | --- | --- | --- |
| 3 months or less |  | 1.82 (1.31, 2.52) | 47 |
| 3 months-6 months or less |  | 1.04 (0.66, 1.62) | 56 |
| 6 months or longer |  |  | NA |
|  |  |  |  |
|  |  |  |  |
|  |  |  |  |
