# Supplementary material for: Social network interventions for health behaviours and outcomes: A systematic review and meta-analysis
Source: PLoS Med. 2019 Sep 3;16(9):e1002890. doi: 10.1371/journal.pmed.1002890 (PMC6719831; doi:10.1371/journal.pmed.1002890)
Supplement: S10 Fig — (DOCX) [file pmed.1002890.s020.docx]

**S10 Fig: Forest plot for subgroup analysis of sexual health outcomes reported at last follow-up: intervention length (3 months or less; 3 months—6 months or less; 6 months—12 months or less; 12 months—18 months or less; greater than 18 months)**

Favours Intervention

Favours Control

| **Intervention length** |  | **Odds ratio (95% CI)** | **I-squared (%)** |
| --- | --- | --- | --- |
| 3 months or less |  |  | NA |
| 3 months-6 months or less |  | 0.77 (0.42, 1.44) | NA |
| 6 months-12 months or less |  |  | NA |
| 12 months-18 months or less |  | 1.36 (1.19, 1.56) | NA |
| 18 months or longer |  | 2.75 (0.74, 10.22) | 94 |
|  |  |  |  |
|  |  |  |  |
|  |  |  |  |
