# Supplementary material for: Social network interventions for health behaviours and outcomes: A systematic review and meta-analysis
Source: PLoS Med. 2019 Sep 3;16(9):e1002890. doi: 10.1371/journal.pmed.1002890 (PMC6719831; doi:10.1371/journal.pmed.1002890)
Supplement: S11 Fig — (DOCX) [file pmed.1002890.s021.docx]

**S11 Fig: Forest plot for subgroup analysis of drug risk outcomes reported at** ≤**six months: intervention length (3 months or less; 3 months—6 months or less; 6 months or longer)**

| **Intervention length** |  | **Odds ratio (95% CI)** | **I-squared (%)** |
| --- | --- | --- | --- |
| 3 months or less |  | 1.98 (0.39, 9.91) | 94 |
| 3 months-6 months or less |  | 1.18 (0.73, 1.91) | 67 |
| 6 months or longer |  |  | NA |
|  |  |  |  |
|  |  |  |  |
|  | Favours Intervention  Favours Control |  |  |
