# Supplementary material for: Social network interventions for health behaviours and outcomes: A systematic review and meta-analysis
Source: PLoS Med. 2019 Sep 3;16(9):e1002890. doi: 10.1371/journal.pmed.1002890 (PMC6719831; doi:10.1371/journal.pmed.1002890)
Supplement: S14 Fig — (DOCX) [file pmed.1002890.s024.docx]

**S14 Fig: Forest plot for subgroup analysis of sexual health outcomes reported at** ≤**six months: participant age (above or below mean age of 32.4 years across studies)**

Favours Intervention

Favours Control

| **Mean age of participants** |  | **Odds ratio (95% CI)** | **I-squared (%)** |
| --- | --- | --- | --- |
| Less than or equal to mean of 32.4 years |  | 1.86 (1.40, 2.48) | 32 |
| Greater than mean of 32.4 years |  | 0.91 (0.64, 1.30) | 43 |
|  |  |  |  |
|  |  |  |  |
|  |  |  |  |
