# Supplementary material for: Social network interventions for health behaviours and outcomes: A systematic review and meta-analysis
Source: PLoS Med. 2019 Sep 3;16(9):e1002890. doi: 10.1371/journal.pmed.1002890 (PMC6719831; doi:10.1371/journal.pmed.1002890)
Supplement: S17 Fig — (DOCX) [file pmed.1002890.s027.docx]

**S17 Fig: Forest plot for subgroup analysis of drug risk outcomes reported at** ≤**six months: participant age (above or below mean age of 32.4 years across studies)**

| **Mean age of participants** |  | **Odds ratio (95% CI)** | **I-squared (%)** |
| --- | --- | --- | --- |
| Less than or equal to mean of 32.4 years |  | 1.08 (0.71, 1.66) | 66 |
| Greater than mean of 32.4 years |  | 1.79 (0.69, 4.66) | 86 |
|  |  |  |  |
|  |  |  |  |

Favours Intervention

Favours Control
