# Supplementary material for: Social network interventions for health behaviours and outcomes: A systematic review and meta-analysis
Source: PLoS Med. 2019 Sep 3;16(9):e1002890. doi: 10.1371/journal.pmed.1002890 (PMC6719831; doi:10.1371/journal.pmed.1002890)
Supplement: S22 Fig — (DOCX) [file pmed.1002890.s032.docx]

**S22 Fig: Forest plot for subgroup analysis of sexual health outcomes reported at last follow-up: participant gender (above or below mean of 45.5% female across studies)**

| **Percentage female participants** | Favours Control  Favours Intervention | **Odds ratio (95% CI)** | **I-squared (%)** |
| --- | --- | --- | --- |
| Less than or equal to mean of 45.5% female |  | 1.28 (1.01, 1.62) | 29 |
| Greater than mean of 45.5% female |  | 7.88 (4.94, 12.57) | NA |
|  |  |  |  |
|  |  |  |  |
|  |  |  |  |
