# Supplementary material for: Social network interventions for health behaviours and outcomes: A systematic review and meta-analysis
Source: PLoS Med. 2019 Sep 3;16(9):e1002890. doi: 10.1371/journal.pmed.1002890 (PMC6719831; doi:10.1371/journal.pmed.1002890)
Supplement: S24 Fig — (DOCX) [file pmed.1002890.s034.docx]

**S24 Fig: Forest plot for subgroup analysis of drug risk outcomes reported at >six months to <12 months: participant gender (above or below mean of 45.5% female across studies)**

Favours Intervention

Favours Control

| **Percentage female participants** |  | **Odds ratio (95% CI)** | **I-squared (%)** |
| --- | --- | --- | --- |
| Less than or equal to mean of 45.5% female |  | 1.40 (0.74, 2.64) | 66 |
| Greater than mean of 45.5% female |  |  | NA |
|  |  |  |  |
|  |  |  |  |
|  |  |  |  |
