# Supplementary material for: Social network interventions for health behaviours and outcomes: A systematic review and meta-analysis
Source: PLoS Med. 2019 Sep 3;16(9):e1002890. doi: 10.1371/journal.pmed.1002890 (PMC6719831; doi:10.1371/journal.pmed.1002890)
Supplement: S28 Fig — (DOCX) [file pmed.1002890.s038.docx]

**S28 Fig: Forest plot for sensitivity analysis of sexual health outcomes reported at last follow-up: Risk of bias**

| **Risk of bias** | Favours Intervention  Favours Control | **Odds ratio (95% CI)** | **I-squared (%)** |
| --- | --- | --- | --- |
| High ROB |  | 1.85 (0.91, 3.74) | 93 |
| Low/unclear ROB |  |  | NA |
|  |  |  |  |
