# Supplementary material for: Social network interventions for health behaviours and outcomes: A systematic review and meta-analysis
Source: PLoS Med. 2019 Sep 3;16(9):e1002890. doi: 10.1371/journal.pmed.1002890 (PMC6719831; doi:10.1371/journal.pmed.1002890)
Supplement: S30 Fig — (DOCX) [file pmed.1002890.s040.docx]

**S30 Fig: Forest plot for sensitivity analysis of drug risk outcomes reported at >six months to <12 months: Risk of bias**

Favours Intervention

Favours Control

| **Risk of bias** |  | **Odds ratio (95% CI)** | **I-squared (%)** |
| --- | --- | --- | --- |
| High ROB |  | 1.09 (0.83, 1.43) | NA |
| Low/unclear ROB |  | 2.13 (1.04, 4.35) | NA |
|  |  |  |  |
|  |  |  |  |
|  |  |  |  |
|  |  |  |  |
