# Supplementary material for: Social network interventions for health behaviours and outcomes: A systematic review and meta-analysis
Source: PLoS Med. 2019 Sep 3;16(9):e1002890. doi: 10.1371/journal.pmed.1002890 (PMC6719831; doi:10.1371/journal.pmed.1002890)
Supplement: S34 Fig — (DOCX) [file pmed.1002890.s044.docx]

**S34 Fig: Forest plot for sensitivity analysis of sexual health outcomes reported at last follow-up: Intention-to-treat**

| **Intention-to-treat** | Favours Intervention  Favours Control | **Odds ratio (95% CI)** | **I-squared (%)** |
| --- | --- | --- | --- |
| ITT analysis |  | 1.65 (1.34, 2.04) | 15 |
| No ITT analysis/unclear |  | 1.45 (1.11, 1.88) | 47 |
|  |  |  |  |
|  |  |  |  |
|  |  |  |  |
