# Supplementary material for: Social network interventions for health behaviours and outcomes: A systematic review and meta-analysis
Source: PLoS Med. 2019 Sep 3;16(9):e1002890. doi: 10.1371/journal.pmed.1002890 (PMC6719831; doi:10.1371/journal.pmed.1002890)
Supplement: S35 Fig — (DOCX) [file pmed.1002890.s045.docx]

**S35 Fig: Forest plot for sensitivity analysis of drug risk outcomes reported at** ≤**six months: intention-to-treat analysis**

Favours Intervention

Favours Control

| **Intention-to-treat** |  | **Odds ratio (95% CI)** | **I-squared (%)** |
| --- | --- | --- | --- |
| ITT analysis |  | 1.32 (0.84, 2.09) | 69 |
| No ITT analysis/unclear |  | 1.35 (0.42, 4.31) | 89 |
|  |  |  |  |
|  |  |  |  |
|  |  |  |  |
|  |  |  |  |
