# Supplementary material for: Social network interventions for health behaviours and outcomes: A systematic review and meta-analysis
Source: PLoS Med. 2019 Sep 3;16(9):e1002890. doi: 10.1371/journal.pmed.1002890 (PMC6719831; doi:10.1371/journal.pmed.1002890)
Supplement: S38 Fig — (DOCX) [file pmed.1002890.s048.docx]

**S38 Fig: Forest plot for sensitivity analysis of sexual health outcomes reported at** ≤**six months: attrition rate**

| **Attrition rate** | Favours Control  Favours Intervention | **Odds ratio (95% CI)** | **I-squared (%)** |
| --- | --- | --- | --- |
| Attrition rate less than 20% |  | 1.78 (1.26, 2.50) | 58 |
| Attrition rate 20% or greater |  | 0.85 (0.62, 1.16) | 0 |
|  |  |  |  |
|  |  |  |  |
|  |  |  |  |
