# Supplementary material for: Social network interventions for health behaviours and outcomes: A systematic review and meta-analysis
Source: PLoS Med. 2019 Sep 3;16(9):e1002890. doi: 10.1371/journal.pmed.1002890 (PMC6719831; doi:10.1371/journal.pmed.1002890)
Supplement: S45 Fig — (DOCX) [file pmed.1002890.s055.docx]

**S45 Fig: Forest plot for sensitivity analysis of sexual health outcomes reported at >6 months to <12 months: Study design**

| **Study design** | Favours Control  Favours Intervention | **Odds ratio (95% CI)** | **I-squared (%)** |
| --- | --- | --- | --- |
| Randomized Controlled Trials and Cluster RCTs |  | 1.63 (1.42, 1.87) | 0 |
| Other study designs |  | 0.85 (0.57, 1.28) | NA |
