# Supplementary material for: Social network interventions for health behaviours and outcomes: A systematic review and meta-analysis
Source: PLoS Med. 2019 Sep 3;16(9):e1002890. doi: 10.1371/journal.pmed.1002890 (PMC6719831; doi:10.1371/journal.pmed.1002890)
Supplement: S46 Fig — (DOCX) [file pmed.1002890.s056.docx]

**S46 Fig: Forest plot for sensitivity analysis of sexual health outcomes reported at last follow-up: Study design**

| **Study design** | Favours Control  Favours Intervention | **Odds ratio (95% CI)** | **I-squared (%)** |
| --- | --- | --- | --- |
| Randomized Controlled Trials and Cluster RCTs |  | 2.12 (0.86, 5.23) | NA |
| Other study designs |  | 1.79 (0.80, 4.03) | 95 |
