# Supplementary material for: Social network interventions for health behaviours and outcomes: A systematic review and meta-analysis
Source: PLoS Med. 2019 Sep 3;16(9):e1002890. doi: 10.1371/journal.pmed.1002890 (PMC6719831; doi:10.1371/journal.pmed.1002890)
Supplement: S51 Fig — (DOCX) [file pmed.1002890.s061.docx]

**S51 Fig: Funnel plot for sexual health outcome measures reported at >six months to <12 months**
